# Hepatic stellate cell-derived microfibrillar-associated protein 2 prevents liver fibrosis by regulating extracellular matrix and inflammation

**DOI:** 10.7150/thno.109771

**Published:** 2025-03-10

**Authors:** Wen Zhang, Wenyue Wu, Ning Zhang, Hong Li, Yameng Sun, Xiaodong Ge, Hui Han, Shuyan Chen, Anjian Xu, Sai Santosh Babu Komakula, Chao Wang, Nithyananthan Subramaniyam, Qi Han, Aiting Yang, Xuzhen Yan, Natalia Nieto, Hong You, Wei Chen

**Affiliations:** 1 Liver Research Center, Beijing Friendship Hospital, Capital Medical University, Beijing 100050, China.; 2 State Key Lab of Digestive Health, Beijing Friendship Hospital, Capital Medical University, Beijing 100050, China.; 3 National Clinical Research Center of Digestive Diseases, Beijing Friendship Hospital, Capital Medical University, Beijing 100050, China.; 4 Experimental and Translational Research Center, Beijing Friendship Hospital, Capital Medical University, Beijing 100050, China.; 5 Beijing Clinical Research Institute, Beijing Friendship Hospital, Capital Medical University, Beijing 100050, China.; 6 Chinese Institutes for Medical Research (CIMR), Beijing 100069, China.; 7 Department of Pathology, University of Illinois Chicago, Chicago, IL 60612, USA.; 8 Division of Gastroenterology and Hepatology, Department of Medicine, University of Illinois Chicago, Chicago, IL 60612, USA.

**Keywords:** Liver fibrosis, extracellular matrix, hepatic stellate cell, macrophage, matrisome

## Abstract

Microfibrillar-associated protein 2 (MFAP-2) is a crucial component of the extracellular matrix (ECM) microfibrils, yet its role in liver fibrosis remains elusive.

**Methods**: Human tissue arrays and mouse models of fibrosis progression and resolution were used to investigate MFAP-2 expression patterns. *Mfap2* deficiency (*Mfap2*^-/-^) or overexpression (ov*Mfap2*) mice were subjected to carbon tetrachloride (CCl_4_) injection or bile duct ligation (BDL) to induce liver fibrosis. Histological, biochemical, bulk, or single-cell RNA-sequencing (scRNA-seq), proteomics to analyze the matrisome, and *in vitro* studies were conducted.

**Results**: MFAP-2 was predominantly enriched in activated hepatic stellate cells (HSCs) and upregulated in advanced liver fibrosis. Although *Mfap2* ablation had minimal impact on collagen deposition during CCl_4_ injection, it significantly delayed fibrosis regression after CCl_4_ cessation. The delayed fibrosis regression due to *Mfap2* deficiency was likely linked to aggravated intrahepatic inflammation, ECM stabilization, and activated focal adhesion signaling in HSCs. Mechanically, inhibiting HSC-derived *Mfap2* enhanced HSC interactions and increased matrisome protein production, while reducing the interaction between HSCs and liver-resident macrophages by decreasing macrophage migration inhibitory factor secretion from HSCs. Additionally, we validated the role of *Mfap2* deletion in liver fibrosis using the BDL mouse model, demonstrating a more pronounced effect on fibrosis progression. Adeno-associated virus vector (serotype 6)-mediated *Mfap2* overexpression in HSCs conferred protection against liver fibrosis in both models.

**Conclusion**: This study reveals the compensatory protective effects of HSC-derived MFAP-2 on liver fibrosis and its underlying mechanisms. Enhancing MFAP-2 in HSCs may therefore benefit patients with liver fibrosis.

## Introduction

Excessive accumulation of extracellular matrix (ECM) during liver fibrosis results in increased matrix stiffness and viscoelasticity, ultimately culminating in cirrhosis and hepatocellular carcinoma 1, 2. While histological fibrosis regression has demonstrated a notable reduction in the occurrence of clinical endpoint events in individuals with liver fibrosis 3, 4, there are currently no approved therapies specifically targeting liver fibrosis. Moreover, addressing the underlying cause alone is often insufficient for alleviating liver fibrosis 5. Therefore, there is an urgent need for therapeutic targets that can either limit ECM deposition or promote its degradation in liver fibrosis.

Microfibrillar-associated protein 2 (MFAP-2) is a component of the ECM microfibrils, initially isolated from elastic tissues 6. Despite its potential for non-covalent interaction with tropoelastin, fibrillins, biglycan, decorin, and the α3 chain of collagen VI, the absence of *Mfap2* in mice does not disrupt the normal structure and function of elastin-rich tissues 7, 8; thus, it is not essential for assembling elastic or other ECM fibrils. Instead, *Mfap2^-/-^* mice have delayed wound healing and bleeding diathesis 9. These findings complicate the role of MFAP-2 in ECM assembly, and promote us to investigate the potential consequences of altered MFAP-2 expression on the recurring injury and healing that result in ECM remodeling during liver fibrosis.

Previous studies conducted by the Mecham group have revealed that the diverse pathological phenotypes observed in *Mfap2^-/-^* mice are closely linked to the upregulation of transforming growth factor (TGF) signaling 7-10. MFAP-2 is known to remove active TGFβ1 from the cellular milieu and inhibit the binding of latent TGFβ1 to the ECM, functioning as an anti-inflammatory molecule. However, a recent study found that MFAP-2 inhibition via lentivirus-mediated shRNA deactivated TGFβ1 signaling in the liver, alleviating liver fibrosis and inflammation in carbon tetrachloride (CCl_4_) mouse models 11. This finding contradicts the established understanding of MFAP-2 as a suppressor of inflammation through the inhibition of TGFβ1 signaling, which requires further comprehensive investigations.

In this study, we employed human liver tissue arrays, liver fibrosis mouse models, MFAP-2 (*Mfap2*) deficiency (*Mfap2*^-/-^) and overexpression (ov*Mfap2*) mice, multiplex immunofluorescence (IF), multi-omics analyses, and *in vitro* studies to explore the role and mechanisms of MFAP-2 during the progression and, particularly, the regression of liver fibrosis across CCl_4_ and bile duct ligation (BDL) mouse models.

## Material and Methods

### Mice

*Mfap2*^-/-^ mice were obtained from Cyagen Bioscience Inc. (Suzhou, China). Exons 2-to-9 of the *Mfap2* gene were deleted using CRISPR/Cas-mediated genome engineering. Heterozygous *Mfap2*^+/-^ mice were cross-bred to generate littermates (*Mfap2*^+/+^) as controls. C57BL/6J mice (6-8 weeks old, male, ~21.0±2.0 g, HFK Bioscience Co. Ltd., Beijing, China) were injected intravenously via the tail vein with 1.8 × 10^11^ viral genomes of the AAV6-CMV-*Mfap2*-HA-EF1a-mNeonGreen-3×FLAG-WPRE vector (OBiO, Shanghai, China) to generate myofibroblast-specific *Mfap2* overexpressed mice (ov*Mfap2*). As a control (Null), the AAV6-CMV-MCS-EF1a-mNeonGreen-3×FLAG-WPRE vector (OBiO) was injected intravenously via the tail vein.

### Induction of liver injury

The CCl_4_ (Innochem, Shanghai, China) was used to induce liver fibrosis progression and resolution. C57BL/6J, *Mfap2*^+/+^,* Mfap2*^-/-^, Null, and ov*Mfap2* mice received intraperitoneal injections twice a week with 12.5% CCl_4_ in mineral oil (MO, 1/7, v/v, Thermo Fisher Scientific, MA, USA) at a dose of 0.01 ml/g of body weight for one week (1W), four weeks (4W), six weeks (6W), eight weeks (8W), or an equal volume of MO as control. Liver fibrosis resolution was achieved after a three- or four-week cessation of CCl_4_ administration (R3W or R4W). Additionally, BDL was performed to induce cholestatic liver fibrosis, as previously reported 12. All mice were sacrificed at the indicated time points, and serum and liver samples were collected for further analyses.

### Human liver tissue array

A paraffin-embedded human liver tissue array, consisting of adjacent normal liver tissues (n = 26), cancer-adjacent liver tissues (n = 4), chronic hepatitis samples (n = 10), and cirrhosis specimens (n = 40), was obtained from US Biomax, Inc. (MD, USA).

### General methodology

Measurement of serum ALT and AST levels, along with H&E staining and Sirius red staining, mRNA and protein isolation, qPCR, Western blot, immunohistochemistry (IHC), and IF staining were performed as reported in our prior study 13. The isolation of mouse primary hepatocytes, macrophages (Møs), and hepatic stellate cells (HSCs) was performed using Percoll (Cytiva, MA, USA) or Nycodenz (AXELL, Stockholm, Switzerland) as previously reported 14, 15.

### Multiplex IF

Multiplex IF staining was performed using a PANO 4-plex IHC Kit (Panovue, Beijing, China) following the manufacturer's instructions. Briefly, paraffin-embedded liver slices (7-μm) were dewaxed, rehydrated, and subjected to antigen retrieval following standard IHC procedures. After blocking with 10% bovine serum albumin, a series of distinct primary antibodies were sequentially applied, followed by incubation with horseradish peroxidase-conjugated secondary antibodies and tyramide signal amplification (TSA). Microwave heat treatment was applied to the slides following each TSA step. Nuclei were counterstained with DAPI after labeling all target antigens.

### Histological image acquisition and semi-quantitation

Histological images were acquired using a 3DHISTECH Panoramic Scanner (3DHISTECH, Budapest, Hungary) or a laser scanning confocal microscope (Olympus, Tokyo, Japan). Semi-quantitative analysis of Sirius Red staining or IHC staining was conducted using Image-Pro Plus software (version 6.0, Media Cybernetics, MD, USA). The liver injury severity was assessed and graded based on H&E staining, employing the Suzuki score system on a scale from 0-to-4 16.

### Active TGFβ1 measurement

The quantitation of active TGFβ1 in mouse liver tissue was conducted using the commercially available LEGEND MAX™ Free Active TGFβ1 ELISA Kit (Biolegend, CA, USA) according to the manufacturer's instructions.

### Cell culture and treatment

The human HSC line LX-2 cells were cultured in Minimum Essential Medium (Procell, Wuhan, China), supplemented with 10% fetal bovine serum (Sigma, MO, USA) and 100 U/mL penicillin and streptomycin (Gibco, NY, USA). The human monocyte THP-1 cells were cultured in RPMI-1640 Complete Medium (Procell). All cells were maintained in a humidified cell culture incubator at 37 °C with a 5% CO_2_ and 95% O_2_ atmosphere. Exponentially growing cells were seeded in 6-well plates. Upon reaching 70-80% confluence, they were transfected with the following substances using X-tremeGENE HP DNA Transfection Reagent (Roche, Basel, Switzerland): pRP[Exp]-EGFP/Puro-EF1A>h*MFAP2*/FLAG plasmid (2.0 μg/mL, Vectorbuilder, Guangzhou, China), Null plasmid (pRP[Exp]-EGFP/Puro-EF1A>ORF_Stuffer, Vectorbuilder, 2.0 μg/mL), human *MFAP2* siRNA (target sequence: ACUGUACGAACACAGAUCUCCTTP, 50 nM, OBiO), or negative control (NC) siRNA (50 nM, OBiO). These transfections were performed with or without stimulation of recombinant human (rh)TGFβ1 (10 ng/mL, MCE, Shanghai, China). After incubation for the indicated times, cells or their conditioned media (CM) were collected for further analysis or co-culture.

### Protein concentration

Proteins in the cell culture medium were concentrated using the Pierce™ Protein Concentrator PES 10K MWCO, 2-6 mL, 24PK (Thermo Fisher Scientific), following the vendor's instructions. The cell culture media were initially loaded into the concentrator sample chamber and placed inside a collection tube. Subsequently, the concentrator sample chamber was positioned in the rotor with appropriate counterbalance and centrifuged at 12,000 g until the desired concentration factor was attained. The concentrated sample from the bottom and center of the sample chamber was carefully aspirated using a pipette tip and utilized for Western blotting analyses.

### Liver decellularization

Liver tissues (~100 mg) from *Mfap2*^-/-^ and *Mfap2*^+/+^ mice injected with CCl_4_ for eight weeks were finely dissected into small pieces, weighed, and then transferred into pre-cooled tubes for decellularization following the method outlined by Baiocchini et al. 17. Initially, plasma proteins were removed by overnight shaking (600 rpm, 4 ℃) in a solution containing 0.5 M NaCl (Sigma), 10 mM Tris base (Solarbio, Beijing, China), and 1X protease inhibitor (Yeasen, Shanghai, China). Following centrifugation, the pellets were incubated with 1% sodium dodecyl sulfate (SDS, Sigma) and 1X protease inhibitor, shaken at 800 rpm overnight at room temperature. The SDS decellularization process was repeated until the liver tissues were completely decellularized. Subsequently, 80% acetone (Sinopharm Chemical Reagent Co., Ltd, Beijing, China) was used to co-incubate with the pellets for 90 minutes to eliminate residual SDS. The quality of decellularized ECM scaffolds was evaluated through Sirius Red staining.

### Proteolytic digestion of liver ECM scaffold

Following our previously described protocol, the liver ECM scaffold underwent in-solution digestion 18. Initially, decellularized ECMs (~5-10 mg of dry weight) were resuspended and treated with 10 mM dithiothreitol (Thermo Fisher Scientific) in an 8M urea solution (Sigma) at 37 °C for 2 hours with continuous agitation for reduction. Subsequently, alkylation was performed by adding a 500 mM iodoacetamide solution (Thermo Fisher Scientific) to reach a final concentration of 25 mM in the urea solution, followed by a 30-minute incubation in the dark at room temperature. For de-glycosylation, the urea solution was diluted to 2 M with 100 mM ammonium bicarbonate (Sigma), and 1,000 U of Peptide-N-Glycosidase F (BioLabs, CA, USA) was added for a 2-hour incubation with continuous agitation. The ECM proteins were then enzymatically digested into peptides through sequential treatment with 1 μg of Lys-C (Wako, VA, USA) for 2 hours, 3 μg of trypsin (Promega, CA, USA) overnight, and an additional 2-hour treatment with 1.5 μg of trypsin the next day, all conducted at 37 ℃ with continuous shaking. The digestion process was terminated by acidification using freshly prepared 50% trifluoroacetic acid (Thermo Fisher Scientific) until the pH dropped to ≤ 2.0. The acidified samples were centrifuged at room temperature for 5 minutes at 15,000 g, and the peptide-containing supernatant was immediately used. Subsequently, the Sep-Pak C18 columns (Waters, MA, USA) were employed for desalting the peptide sample. The column was activated with 100% acetonitrile (Sigma), equilibrated with 0.1% formic acid (Sigma), and loaded with the peptide solution. Impurities were removed by washing with 0.1% formic acid, and elution was performed using 70% acetonitrile. The eluted fractions were freeze-dried in a vacuum freeze dryer, reconstituted in 2% acetonitrile/0.1% formic acid, and quantified using the Pierce BCA Protein Assay Kit (Thermo Fisher Scientific) following the manufacturer's guidelines.

### Label-free liquid chromatography-tandem mass spectrometry (LC-MS/MS) analysis

The Nanoflow LC-MS/MS analysis employed a quadrupole Orbitrap mass spectrometer (Orbitrap Eclipse, Thermo Fisher Scientific), directly interfaced with an EASY nLC 1200 ultra-high-pressure system (Thermo Fisher Scientific) through a nano-electrospray ion source. Peptide samples, at 1 μg per injection, were loaded onto a 25 cm analytical column (150-μm inner diameter, filled with ReproSil-Pur C18-AQ 1.9-µm silica particles; Beijing Qinglian Biotech Co., Ltd, Beijing, China) and eluted using a solvent gradient. This gradient initiated at 6% and increased to 12% over 15 minutes, followed by an increase from 12% to 30% over the next 48 minutes, further rising to 40% for 10 minutes, and finally reaching a 10-min purge at 95% solvent, all at a flow rate of 300 nL/minutes (comprising 80% acetonitrile and 0.1% formic acid). The entire run lasted 85 minutes, during which a specially designed oven maintained the column at a constant temperature of 60°C. The MS instrument operated in data-dependent acquisition mode, acquiring MS spectra in the Orbitrap mass analyzer at a resolution of 120,000 over the 350-2000 m/z range, with an automatic gain control target of 4E^5^ and a maximum ion injection time of 50 milliseconds. Following higher-energy collisional dissociation at a normalized collision energy of 30%, MS/MS spectra were captured in the Orbitrap at a resolution of 15,000, with an automatic gain control target of 5E^4^ and a maximum ion injection time of 22 milliseconds.

### MS data preprocessing

The raw data from MS was preprocessed using the Proteome Discoverer suite (version 2.4, Thermo Fisher Scientific). Tandem mass spectra were searched against the UniProt database (https://www.uniprot.org/) using the Sequest HT search engine with specific parameters: fully tryptic specificity, up to two missed cleavages allowed, a minimum peptide length of 6, fixed carbamidomethylation of cysteine residues (+57.02146Da), variable modifications for oxidation of methionine residues (+15.99492Da), a precursor mass tolerance of 15 ppm, and a fragment mass tolerance of 0.02Da for MS2 spectra collected in the Orbitrap. Peptide spectral matches and peptides were filtered to ensure a false discovery rate (FDR) < 1% using a percolator. Following spectral assignment, peptides were assembled into proteins and further refined based on the combined probabilities of their constituent peptides to achieve a final FDR of < 1%. The top matching protein or 'master protein' was determined to contain the highest count of unique peptides and the lowest percent peptide coverage. Unique and razor (i.e., parsimonious) peptides were utilized for quantification. Matrisome protein levels identified via LC-MS/MS were compared between *Mfap2*^-/-^ and *Mfap2*^+/+^ mice.

### Bulk RNA-seq analysis

Bulk RNA-seq analysis was conducted on frozen livers from CCl_4_-treated *Mfap2*^+/+^ and *Mfap2*^-/-^ mice (8W and R4W, n = 4 for each group of mice) as previously described 19. Initially, total RNA was extracted from mouse frozen liver tissues using the RNA simple Total RNA kit (Fastagen, Shanghai, China) following the manufacturer's protocol. Subsequently, poly(A) mRNAs were enriched using magnetic oligo (dT) beads (Invitrogen, CA, USA). According to the guidelines, cDNA libraries were constructed using the NEBNext Ultra RNA Library Prep Kit (New England Biolabs, Hitchin, UK) or the Illumina VAHTS® Universal V6 RNA-seq Library Prep Kit (Vazyme, Nanjing, China). 125 bp paired-end libraries from 8W mice were sequenced on the Illumina HiSeq2500 platform (Biomarker Technologies Co., Ltd, Beijing, China). 150-bp paired-end libraries from R4W mice were sequenced on the Illumina NovaSeq 6000 platform (Shanghai NextCODE Co., Ltd, Shanghai, China). The HISAT software 20 aligned clean reads to the mouse genome (mm10). Read counts and FPKM values for each identified gene were calculated using Cufflinks 21. Differentially expressed genes in livers from *Mfap2*^+/+^ and *Mfap2*^-/-^ mice were analyzed using student's *t*-test and fold change (FC). FC > 1.5 were set as statistically significant criteria. The enriched Kyoto Encyclopedia of Genes and Genomes (KEGG) pathways of the significantly downregulated or upregulated genes were determined using the DAVID web tool (https://david.ncifcrf.gov/). KEGG pathways with a Benjamini-corrected *p* value < 0.05 were visualized using the *ggplot2* package. The *mMCPcounter* package 22 was used to estimate the abundance of infiltrating immune and stromal cell populations in mouse livers based on the bulk RNA-seq data.

### Library preparation for scRNA-seq and sequencing

scRNA-seq of liver non-parenchymal cells (NPCs) isolated from mice injected with mineral oil (control), CCl_4_ (peak fibrosis, four weeks), and during fibrosis resolution (1-week recovery) was conducted using the NovaSeq 6000 system (University of Illinois at Urbana-Champaign DNA Sequencing Laboratory, IL, USA). The methodology for *in vivo* isolation of NPCs and scRNA-seq has been previously described in our prior study 19. Additionally, scRNA-seq was performed on liver NPCs isolated from CCl_4_-injected *Mfap2*^+/+^ and *Mfap2*^-/-^ mice (eight weeks) by Shanghai OE Biotech. Co. Ltd. (Shanghai, China). To isolate the NPCs, anesthetized mice underwent a slow infusion of ice-cold PBS through the hepatic portal vein to remove circulating red blood cells. Subsequently, the livers were carefully shredded on ice into small pieces (less than 1 mm cubic) and placed in a gentleMACS C tube (Miltenyi Biotec, Bergisch Gladbach, Germany) containing a solution of digestive enzymes (Mouse Liver Dissociation Kit; Miltenyi Biotec). The liver was then homogenized using a GentleMACS™ dissociation machine (Miltenyi Biotec). The resulting cell mixture was filtered through a 40-µm cell strainer (Miltenyi Biotec). After centrifugation (300 g, 5 minutes), the cell pellet was resuspended in a 40% Percoll solution (Cytiva). Following a second round of centrifugation (600 g, 15 minutes), the cell pellet was collected and then processed for GEM generation and barcoding, post-GEM-RT cleanup and cDNA amplification, and 3ʹ gene expression library construction. These steps involved the use of Chromium Single Cell 3' Library & Single Cell 3' v3 Gel Beads (10× Genomics, CA, USA), DynaBeads® MyOne^TM^ Silane Beads (Life Technologies, MA, USA), SPRIselect Reagent Kit (Life Technologies), Qubit dsDNA Assay Kit (Life Technologies), and Agilent High Sensitivity DNA Kit (Agilent, CA, USA), according to the manufacturer's protocols. Finally, the cDNA libraries were sequenced using the MGISEQ-2000 sequencing platform (MGI Tech, Shenzhen, China).

### Preprocessing and scRNA-seq data analysis

The raw scRNA-seq data underwent preprocessing using 10× Genomics software *CellRanger* (version 3.1.0), encompassing sample demultiplexing, alignment to the reference genome (mm10), filtering, and gene-level unique molecular identifier counting. Subsequently, the *Seurat* package was employed for quality control. Specifically, cells with nFeature_RNA > 1000 & percent_mito < 25% & percent_ribo > 3% & percent_hb < 1% were retained, and genes expressed in more than 5 cells were retained. The *FindVariableGenes* function in the *Seurat* package was used to identify highly variable genes, which were then subjected to principal component analysis for dimension reduction and visualized in two-dimensional space using uniform manifold approximation and projection for dimension reduction plot. Cell types were annotated according to the known marker genes. The *FindMarkers* function in the *Seurat* package was employed to identify marker genes that exhibited differential upregulation in each cell type compared to other cell groups (adjusted *p*-value < 0.05 and Log_2_[FC] > 0.5). To gain insights into biological pathways, KEGG pathway enrichment analysis was performed on the top 1,000 genes with the highest abundance using the* ClusterProfiler* package 23. Lastly, the *CellChat* package 24 was utilized to analyze cell-to-cell interactions based on the expression of specific ligands and receptors.

### Publicly available dataset analysis

Transcriptomic profiles associated with human or mouse liver fibrosis were publicly available from the GEO database. The datasets GSE84044 25, GSE149601 26, and GSE193066 27 include gene expression profiles derived from human non-fibrotic and fibrotic liver tissues with various etiologies, including hepatitis B virus, hepatitis C virus, or metabolic dysfunction-associated steatotic liver disease. Datasets GSE55747 and GSE74605 consist of gene expression profiles derived from mouse livers treated with CCl_4_ or thioacetamide. *MFAP2* gene expression, detected by microarray or bulk RNA-seq, underwent normalization using the robust multichip average algorithm 28 or was scaled into fragments per kilobase per million (FPKM), respectively. Additionally, GSE145086 29 and GSE233751 19, 30, which contain 10× Genomics scRNA-seq data of isolated NPCs from normal or CCl_4_-induced mouse fibrotic livers, were analyzed using *Seurat* package 31 to identify the cellular sources of liver *Mfap2* gene expression.

### Clinical relevance analysis of human liver MFAP2 expression

Liver transcriptomic profiles were obtained from 15 treatment-naïve patients with chronic HBV infection and a baseline Ishak score of ≥ 4, sourced from our previously published dataset (PRJCA010948) 19 as well as our unpublished transcriptomic dataset (available upon request). These patients underwent paired liver biopsies at baseline and after 78 weeks of antiviral treatment. The regression of liver fibrosis was histologically assessed as previously defined 19. After correcting for batch effects using the *ComBat* package 32 and extracting *MFAP2* gene expression levels, we analyzed the relationship between baseline liver *MFAP2* expression and fibrosis regression following HBV suppression.

### Ethical guidelines

Mice were housed and bred in a specific pathogen-free grade animal facility, maintained at a temperature of 23±2℃, under a 12-hour light-dark cycle, and provided with standard chow and water *ad libitum*. The Ethics Committee of Beijing Friendship Hospital, Capital Medical University, and the University of Illinois Chicago IACUC office approved studies involving mice. All *in vivo* mouse experiments were conducted per the ARRIVE guidelines 33. Written informed consent was obtained from all enrolled patients prior to screening. The study protocols were approved by the Ethics Committees of all participating centers (2016-P2-021-04, 2018-P2-106-05) and adhered to the principles of the Declaration of Helsinki.

### Data availability statement

Raw and processed data from bulk RNA RNA-seq or scRNA-seq have been deposited in the GEO database under accession numbers GSE272283 and GSE272284. Raw MS data and Proteome Discoverer output files containing protein identifications have been submitted to the ProteomeXchange consortium through the PRIDE partner repository with the dataset identifier PXD053782. Detailed information regarding the publicly available bulk and scRNA-seq datasets used in this study is presented in **Table S1**. Details of antibodies, chemicals, recombinant proteins, commercial kits and reagents, cell lines, organisms/strains, primers for qPCR, adeno-associated virus (serotype 6, AAV6) vectors, shRNAs, siRNAs, and the software used in this study are listed in **Table S2**.

### Statistical analysis

Continuous variables are expressed as mean ± standard error (SEM). Student's *t*-test or Mann-Whitney *U*-test was utilized to compare continuous variables between groups. One-way ANOVA, followed by the least significant difference test, was employed to compare continuous variables among three or more groups. Categorical variables were presented as percentage and compared by Chi-square test or Chi-square trend test between any two groups. A *p*-value < 0.05 was considered statistically significant. All statistical analysis was performed using the GraphPad Prism version 9 (GraphPad Software, Inc., CA, USA) and R 4.2.1 software (https://www.r-project.org/).

## Results

### MFAP-2 is predominantly enriched in activated HSCs and upregulated in advanced fibrotic livers of humans and mice

**Figure 1:**
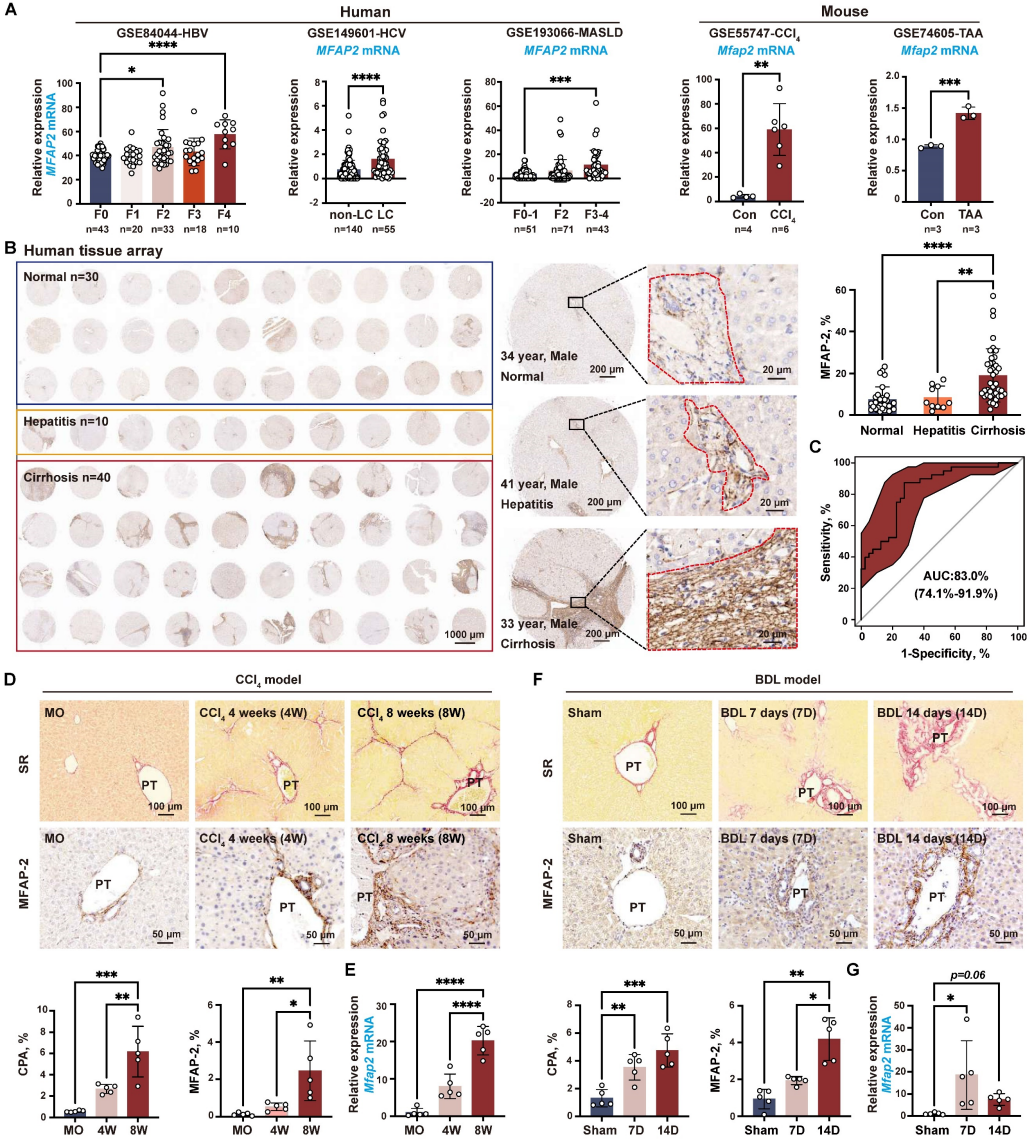
** MFAP-2 is elevated in fibrotic livers from humans and mice.** (**A**) Analysis of *MFAP2/Mfap2* gene expression using publicly available datasets. The sample size for each group is indicated below the respective bar. (**B**) IHC and quantification of MFAP-2 expression in normal (n = 30), hepatitis (n = 10), and cirrhosis (n = 40) livers from a human tissue array. (**C**) ROC analysis based on MFAP-2 expression in liver slices from the human tissue array. (**D-G**) Sirius Red staining, IHC, and qPCR analyses of MFAP-2 (*Mfap2*) expression in liver tissues from CCl_4_ and BDL mouse models (n = 5/group). Data are expressed as mean ± SEM. *p < 0.05, **p < 0.01, ***p < 0.001, and ****p < 0.0001. CPA: collagen proportional area; LC: liver cirrhosis; MO: mineral oil; non-LC: non-liver cirrhosis; PT: portal tract; SR: Sirius Red staining; TAA: thioacetamide.

To investigate the expression pattern of MFAP-2 in liver fibrosis, we first analyzed publicly available transcriptomic datasets. As depicted in **Figure 1A**, *Mfap2* was significantly upregulated in fibrotic livers compared to non-fibrotic livers from both humans and mice, irrespective of etiology. Immunostaining of a human liver tissue array showed a nearly twofold increase in MFAP-2 expression in cirrhotic livers compared to normal or hepatitis livers (**Figure 1B**). Notably, MFAP-2 expression demonstrated strong discriminatory power to effectively distinguish fibrotic from non-fibrotic livers, with an area under the curve of 0.83 (**Figure 1C**). We further validated the expression pattern of MFAP-2 in mouse models of liver fibrosis (CCl_4_ and BDL). Our results indicated that both *Mfap2* gene expression and MFAP-2 protein levels were significantly elevated, particularly in advanced liver fibrosis (**Figure 1D-G**), as seen in humans. Moreover, MFAP-2 protein expression showed a strong correlation with liver fibrosis induced by BDL operation (r = 0.76, *p* < 0.01) and CCl_4_ injection (r = 0.66, *p* < 0.01) (**Figure S1**).

**Figure 2:**
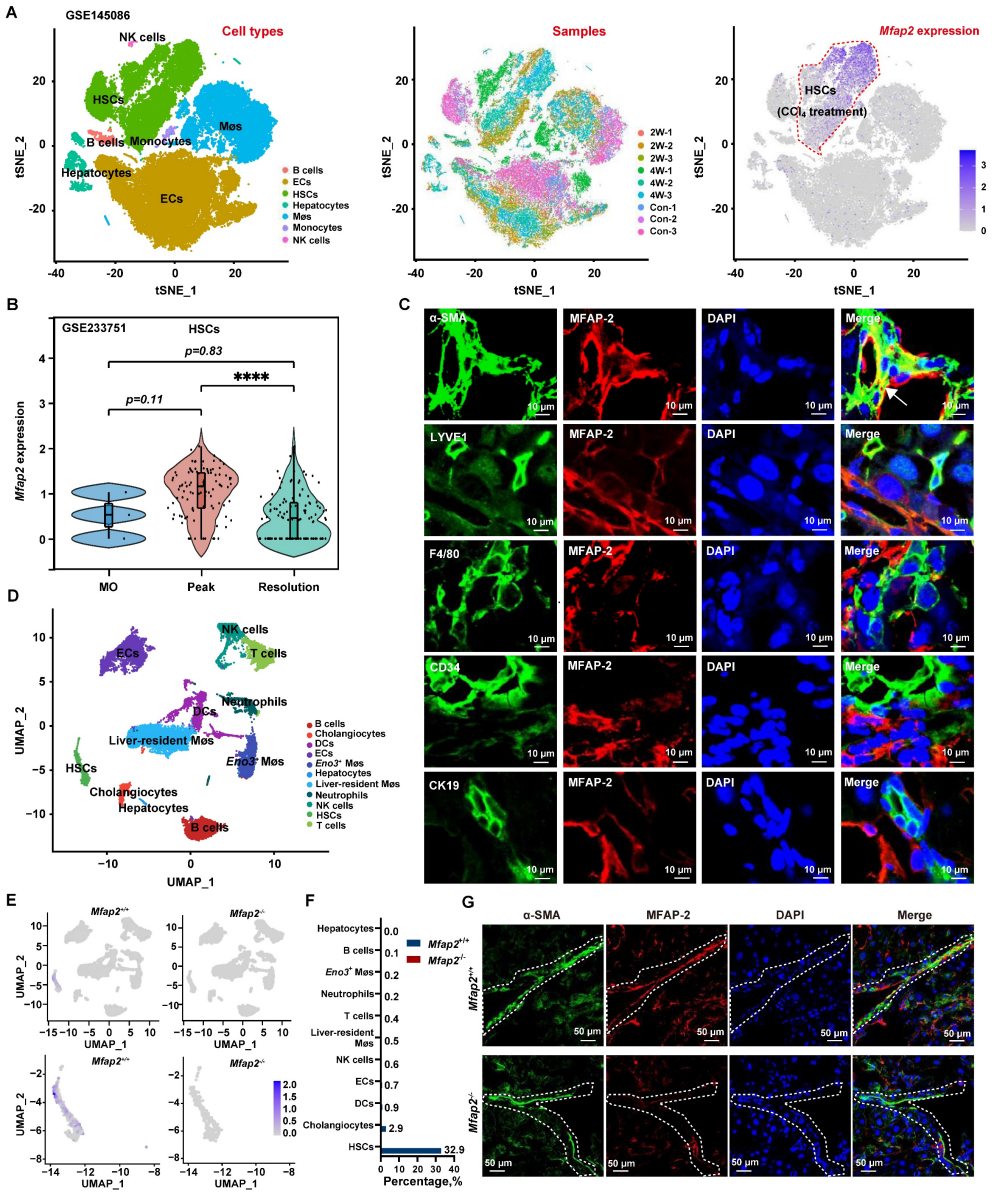
** MFAP-2 is enriched and increased in activated HSCs.** (**A**) scRNA-seq analysis of *Mfap2* gene expression in CCl_4_-injected mouse livers, using the publicly available dataset GSE145086. The t-SNE plots, displayed from left to right, show cell types, samples (Con: control; 2W: two weeks; 4W: four weeks), and *Mfap2* gene expression. (**B**) Comparison of *Mfap2* gene expression in HSCs from control (MO), peak liver fibrosis, and fibrosis regression in mice, based on our published scRNA-seq data (GSE233751). (**C**) Immunofluorescent staining of α-SMA (green), LYVE1 (green), F4/80 (green), CD34 (green), CK19 (green), MFAP-2 (red), and DAPI (nucleus, blue) in liver tissues from CCl_4_-injected mice. Co-localization is indicated in yellow. (**D-F**) UMAP plots showing eleven annotated cell types along with *Mfap2* gene expression, and a comparison of the proportion of *Mfap2*^+^ liver cells. (**G**) Immunofluorescent staining of MFAP-2 (red), α-SMA (green), and DAPI (nucleus, blue) in liver tissues from *Mfap2*^+/+^ and *Mfap2*^-/-^ mice, with co-localization shown in yellow. Data are expressed as mean ± SEM. ****p < 0.0001.

Previous studies have shown that MFAP-2 is mainly expressed in fibroblasts within adipose tissue and the dermis 34, 35; thus, we investigated whether fibroblasts were the primary source of MFAP-2 in the liver. To this end, we analyzed the publicly available scRNA-seq dataset (GSE145086) 29 and our previously published scRNA-seq dataset (GSE233751) 19, 30 of NPCs from livers with CCl_4_-induced liver fibrosis. As anticipated, *Mfap2* was abundantly expressed in HSCs from CCl_4_-induced liver fibrosis but showed a notable decrease during fibrosis regression (**Figure 2A-B**).

Subsequent co-localization studies revealed that α-SMA^+^ HSCs were the main cellular source of MFAP-2 expression, while LYVE1^+^ endothelial cells and F4/80^+^ Møs displayed minimal MFAP-2 expression (**Figure 2C**). To further pinpoint the cellular source of MFAP-2, we performed scRNA-seq on NPCs from *Mfap2*^-/-^ and *Mfap2*^+/+^ mice injected with CCl_4_ for eight weeks (**Figure S2**). After quality control and filtering, clustering of 19,295 high-quality cells identified eleven cell lineages based on established cell makers (**Figure 2D**). We found that *Mfap2* was predominantly expressed in 32.9% of HSCs and minimally expressed in other liver cells from *Mfap2*^+/+^ mice injected with CCl_4_ for eight weeks; while its expression was absent in *Mfap2*^-/-^ mice (**Figure 2E-F**). Co-localization studies corroborated the findings from the scRNA-seq analysis (**Figure 2G**). These findings indicate that MFAP-2 is predominantly expressed and upregulated in activated HSCs within advanced fibrotic livers, but its role in the pathogenesis of liver fibrosis remains unknown.

### Despite Mfap2 ablation shows minimal impact on collagen deposition during CCl_4_ injection, it delays fibrosis regression after CCl_4_ cessation

**Figure 3:**
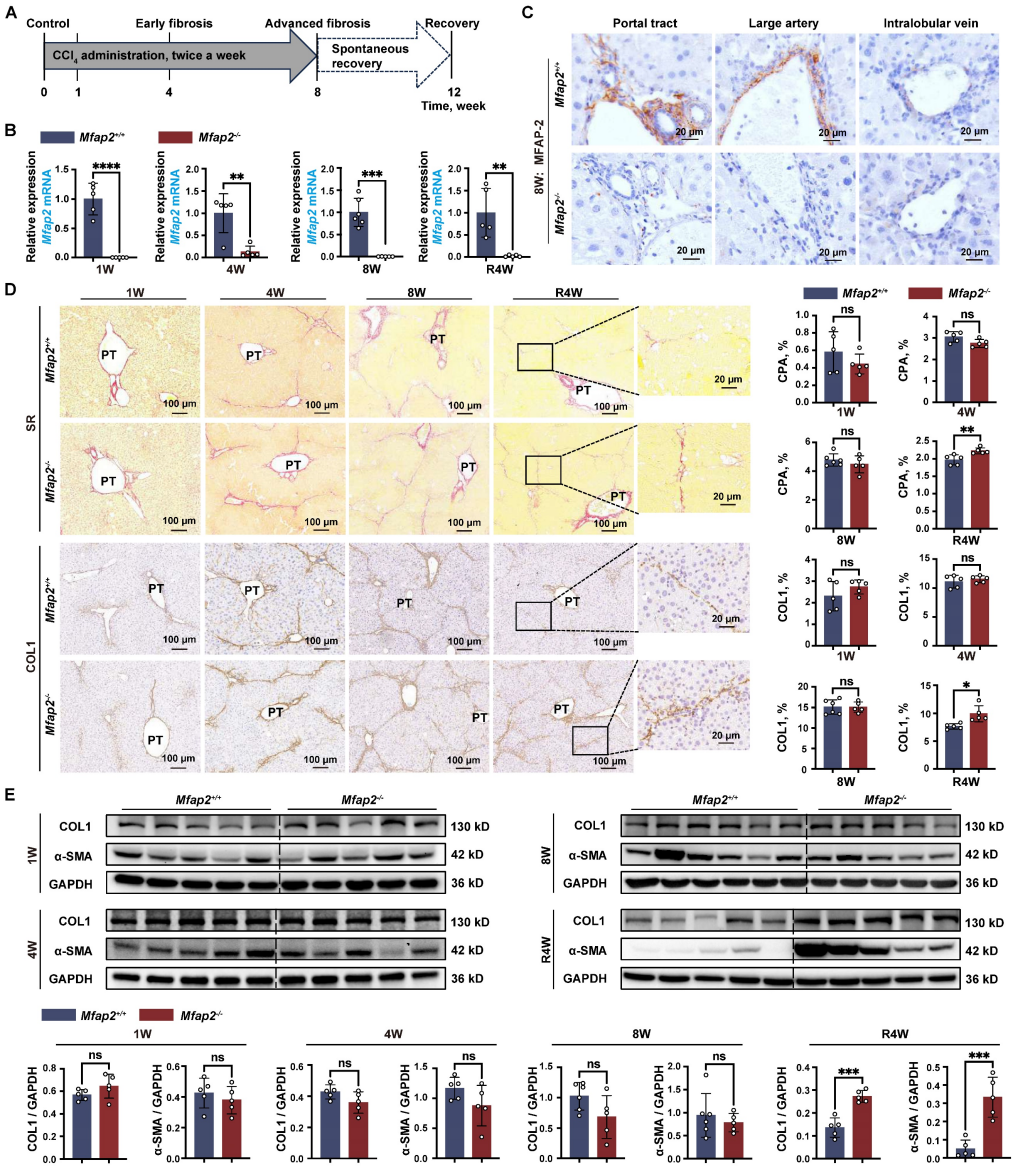
***Mfap2*^-/-^ mice show slower fibrosis regression after CCl_4_ cessation.** *Mfap2*^-/-^ and *Mfap2*^+/+^ mice were injected with CCl_4_ for one (1W), four (4W), or eight (8W) weeks, followed by a cessation period of four weeks (R4W). (**A**) Schematic diagram illustrating the model of CCl_4_-induced liver fibrosis and subsequent resolution. (**B**) qPCR analysis of *Mfap2* gene expression (n = 5-6/group). (**C**) IHC analysis of MFAP-2 expression. (**D**) Sirius Red staining of liver sections and IHC analysis of COL1 expression (n = 5-6/group). (**E**) Immunoblotting analysis of COL1 and α-SMA expression (n = 5-6/group). Data are expressed as mean ± SEM. *p < 0.05, **p < 0.01, ***p < 0.001, and ****p < 0.0001. ns: not significant; PT: portal tract.

Given that activated HSCs were the main MFAP-2-producing cells in the liver, we used *Mfap2*^-/-^ mice to explore the role of HSC-derived MFAP-2 in liver fibrosis. Mice were intraperitoneally injected with CCl_4_ twice a week for one, four, or eight weeks to induce acute liver injury, early fibrosis, and advanced fibrosis, respectively (**Figure 3A**). Although MFAP-2 (*Mfap2*) was almost deleted in the livers of *Mfap2*^-/-^ mice at all time points (**Figure 3B-C**), continuous CCl_4_ injection had minimal effects on fibrosis progression, as indicated by comparable liver-to-body weight ratio, serum ALT and AST levels, ECM collagen deposition, and COL1 and α-SMA expression between *Mfap2*^-/-^ and *Mfap2*^+/+^ mice injected with CCl_4_ for one, four, or eight weeks (**Table S3** and **Figure 3D-E**).

Given the well-established role of MFAP-2 as an ECM protein 6 and its upregulation in activated HSCs, we hypothesized that *Mfap2* ablation could change the ECM, thereby affecting the regression of liver fibrosis after CCl_4_ cessation. To evaluate this, both *Mfap2*^+/+^ and *Mfap2*^-/-^ mice that had been injected with CCl_4_ for eight weeks were allowed to recover. After a four-week recovery period following CCl_4_ cessation, the liver-to-body weight ratio and serum ALT and AST levels remained comparable between *Mfap2*^-/-^ and *Mfap2*^+/+^ mice (**Table S3**). Surprisingly, immunostaining and morphometric analysis of COL1 revealed that *Mfap2*^-/-^ mice exhibited more pronounced, thicker, and continuous collagen fibers in the liver septal areas compared to *Mfap2*^+/+^ mice (**Figure 3D**), suggesting a delayed fibrosis regression in *Mfap2*^-/-^ mice. Moreover, intrahepatic COL1 expression was found to be two-fold higher, and α-SMA expression was 6.7-fold higher in *Mfap2*^-/-^ mice than those in *Mfap2*^+/+^ mice after four weeks of resolution (**Figure 3E**). To precisely evaluate the role of *Mfap2* in liver fibrosis regression, we conducted bulk RNA-seq on regressive liver tissues from both groups of mice. As illustrated in **Figure S3**, the upregulated genes (n = 468) in *Mfap2*^-/-^ mice were predominantly linked to immune and FA signaling-related pathways when compared to *Mfap2*^+/+^ mice; while the downregulated genes (n = 84) in *Mfap2*^-/-^ mice did not show significant enrichment in any pathways. These findings precisely highlight that *Mfap2* ablation evidently delays liver fibrosis regression.

### Mfap2 deficiency-induced intrahepatic inflammation aggravation contributes to the delayed fibrosis regression after CCl_4_ cessation

**Figure 4:**
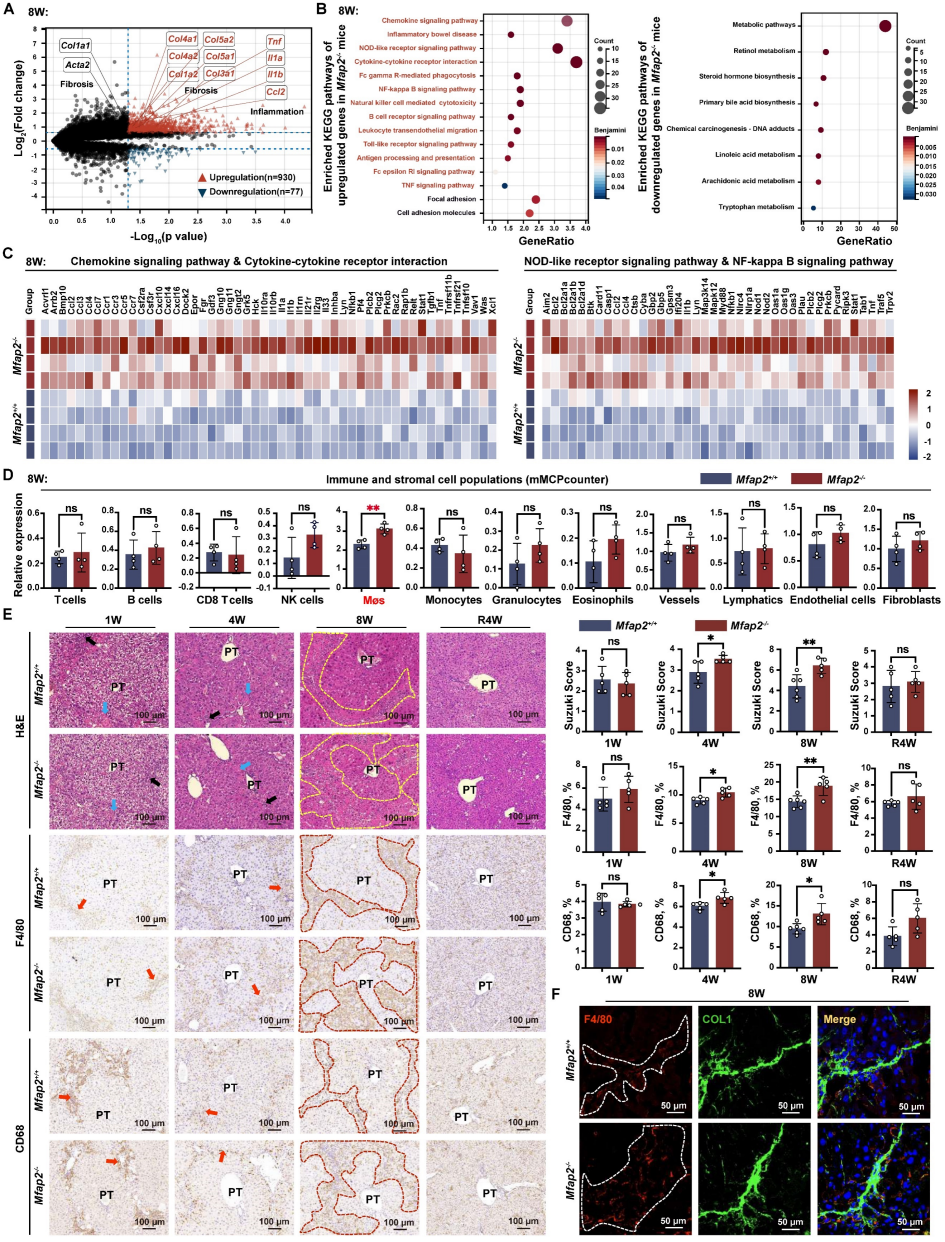
***Mfap2*^-/-^ mice exhibit increased intrahepatic inflammation.** *Mfap2*^-/-^ and *Mfap2*^+/+^ mice were injected with CCl_4_ for one (1W), four (4W), or eight (8W) weeks, followed by a cessation period of four weeks (R4W). (**A**) Volcano plot illustrating differentially expressed genes (n = 4/group; *p* < 0.05 & FC>1.5). (**B**) Significantly enriched KEGG pathways based on upregulated or downregulated genes (Benjamini-corrected *p* < 0.05). (**C**) Heatmaps displaying gene expression in representative immune-related KEGG pathways. (**D**) Comparisons of liver immune and stromal cell populations (n = 4/group). (**E**) H&E staining of liver slices and IHC analysis of F4/80 or CD68 expression (n = 5-6/group). (**F**) Immunofluorescent staining of COL1 (green), F4/80 (red), and DAPI (nucleus, blue). Data are expressed as mean ± SEM. *p < 0.05, **p < 0.01. ns: not significant; PT: portal tract.

To elucidate the underlying cause of the delayed fibrosis regression observed in *Mfap2^-/-^* mice, we performed bulk RNA-seq analysis on the livers of *Mfap2*^-/-^ and *Mfap2*^+/+^ mice that had been injected with CCl_4_ for eight weeks. This analysis unveiled that the absence of MFAP-2 did not significantly alter the gene expression of *Col1a1* and *Acta2*, but notably increased the expression of several other collagen-encoding genes (*Col1a2*, *Col3a1*, *Col4a1*, *Col4a2*, *Col5a1*, *Col5a2*) as well as pro-inflammatory genes (*Tnf*, *Il1a*, *Il1b*, *Ccl2*), which were further validated by qPCR analysis (**Figure 4A** and **S4A**). Overall, a total of 930 genes were found to be upregulated in the livers of *Mfap2*^-/-^ mice compared to *Mfap2*^+/+^ mice after injecting CCl_4_ for eight weeks, with a predominant enrichment in immune-related pathways (i.e., chemokine signaling pathway, cytokine-cytokine receptor interaction, NOD-like receptor signaling pathway, NF-kappa B signaling pathway); in contrast, only 77 genes were downregulated in the livers of *Mfap2*^-/-^ mice, primarily associated with metabolic signaling (**Figure 4A-C**). These results further underscore that *Mfap2* deletion aggravates intrahepatic inflammation while exacerbating metabolic dysfunction at the molecular level.

Subsequently, we employed the *mMCPcounter* algorithm 22 to deconvolute and compare the populations of liver stromal and immune cells between *Mfap2*^+/+^ and *Mfap2*^-/-^ mice injected with CCl_4_ for eight weeks, based on the bulk RNA-seq data. Notably, only Møs showed a significant increase in *Mfap2*^-/-^ mice (**Figure 4D**). Analysis of the Suzuki score of the H&E-stained sections, along with IHC or immunoblotting of F4/80 or CD68, demonstrated that *Mfap2* deficiency in activated HSCs led to increased infiltration of Møs into the livers of mice subjected to chronic CCl_4_ injury (**Figure 4E** and** S5A**). Co-localization studies further confirmed that *Mfap2* ablation exacerbated intrahepatic inflammation, as evidenced by the increased presence of F4/80^+^ Møs surrounding the collagenous fibers (**Figure 4F**). In summary, the loss of *Mfap2* worsens intrahepatic inflammation, thereby hindering the regression of liver fibrosis.

### Mfap2 deletion induces ECM stabilization and activates focal adhesion (FA) signaling in HSCs, impeding the spontaneous fibrosis regression

**Figure 5:**
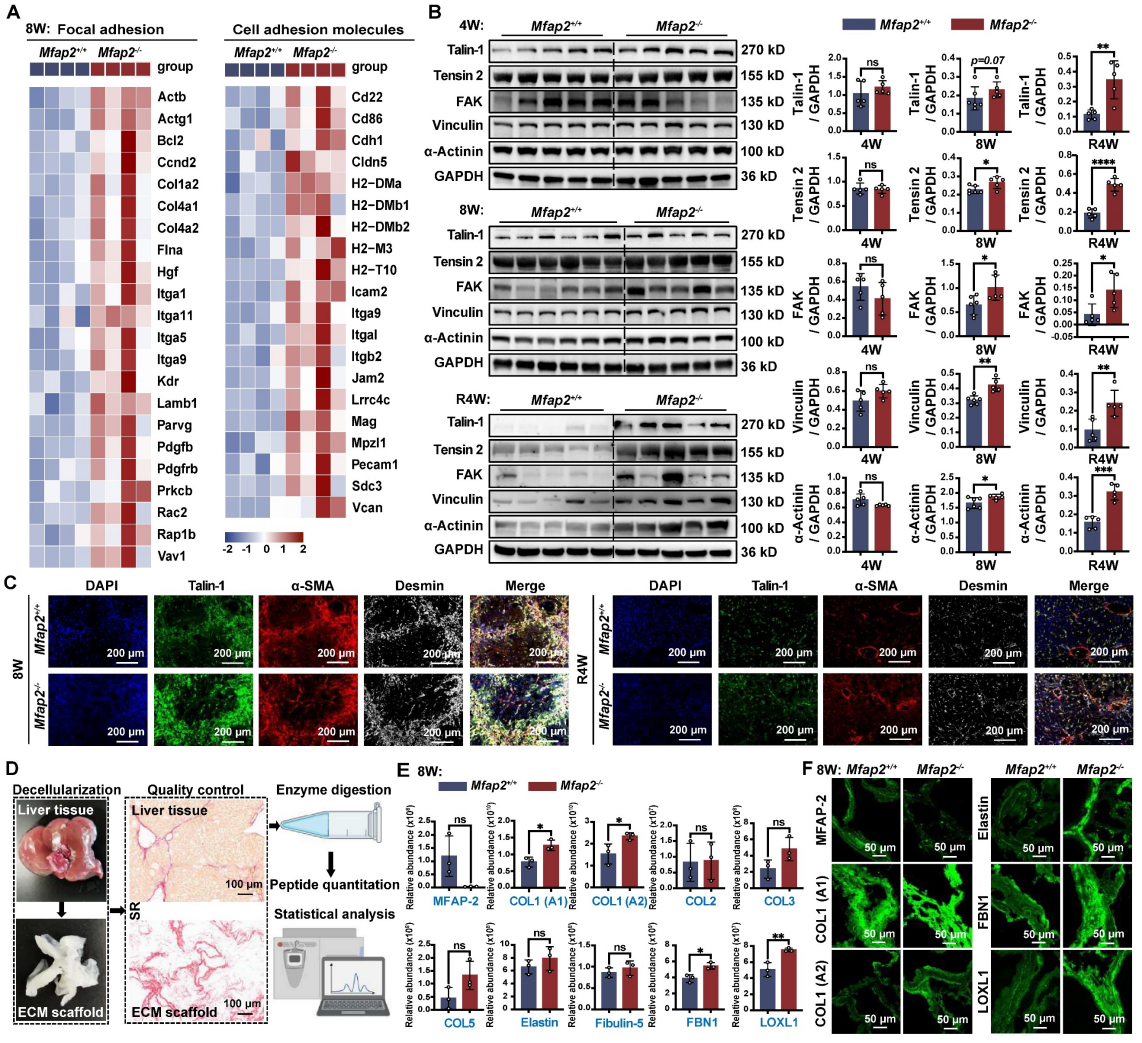
***Mfap2* deletion activates FA signaling in HSCs and stabilizes the ECM following CCl_4_ injection.** *Mfap2*^-/-^ and *Mfap2*^+/+^ mice were injected with CCl_4_ for four (4W) or eight (8W) weeks, followed by a cessation period of four weeks (R4W). (**A**) Heatmaps displaying gene expression of molecules involved in FA and cell adhesion signaling. (**B**) Immunoblotting analysis of FA signaling markers (n = 5-6/group). (**C**) Multiplex IF staining of Talin-1 (green), α-SMA (red), Desmin (grey), and DAPI (nucleus, blue). Co-localization of Talin-1 and α-SMA is indicated in yellow. (**D**) Workflow for liver decellularization, proteolytic digestion, and quantification by MS. (**E**) Comparisons of insoluble collagens and elastic fiber assembly-related ECM components in the decellularized ECM scaffolds (n = 3/group). (**F**) Immunofluorescent staining of MFAP-2, COL1 (A1), COL1 (A2), Elastin, FBN1, and LOXL1 in decellularized ECM scaffolds. Data are expressed as mean ± SEM. *p < 0.05, **p < 0.01, ***p < 0.001, ****p < 0.0001. ns: not significant.

In addition to the infiltration of Møs, the bulk RNA-seq analysis also revealed that FA signaling and cell adhesion molecules were more activated in the livers of *Mfap2*^-/-^ mice than in *Mfap2*^+/+^ mice after eight weeks of CCl_4_ injection (**Figure 5A**). To further investigate this, we performed immunoblotting and confirmed that the lack of *Mfap2* induced the activation of FA signaling, as evidenced by increased expression of Tensin-2, FAK, Vinculin, and α-Actinin in the livers from *Mfap2*^-/-^ mice at eight weeks of CCl_4_ injection (**Figure 5B**). Notably, after four weeks of fibrosis regression, the difference in the activation of liver FA signaling became more pronounced (**Figure 5B**). Moreover, co-localization studies demonstrated that Talin-1, a crucial component of FAs 36, co-localized with α-SMA^+^ Desmin^+^ HSCs, suggesting that the activation of FA signaling following *Mfap2* deletion mainly occurs in activated HSCs (**Figure 5C**).

Since increased FA signaling is often a consequence of ECM remodeling 37, its activation suggests the assembly of a more stable ECM scaffold resulting from *Mfap2* ablation. To investigate this, we decellularized the liver, performed proteolytic digestion, and quantified the components using MS. This analysis allowed us to identify the insoluble matrisome members present in the liver ECM scaffolds of *Mfap2*^-/-^ and *Mfap2*^+/+^ mice after eight weeks of CCl_4_ injection (**Figure 5D**). As shown in **Figure 5E-F**, MFAP-2 levels were nearly undetectable, while the levels of insoluble COL1, FBN1, and LOXL1 significantly increased in the liver ECM scaffolds of *Mfap2*^-/-^ mice following CCl_4_ treatment. Given that LOXL1 is responsible for forming covalent cross-links that stabilize collagen fibrils 38, 39, we speculate that *Mfap2* deletion increases the expression and secretion of ECM components, including LOXL1 from HSCs, which likely contributes to ECM stabilization and the subsequent activation of FA signaling.

### Loss of Mfap2 enhances ECM stabilization by accelerating the production of matrisome proteins, while simultaneously exacerbating intrahepatic inflammation by reducing macrophage migration inhibitory factor (MIF)

**Figure 6:**
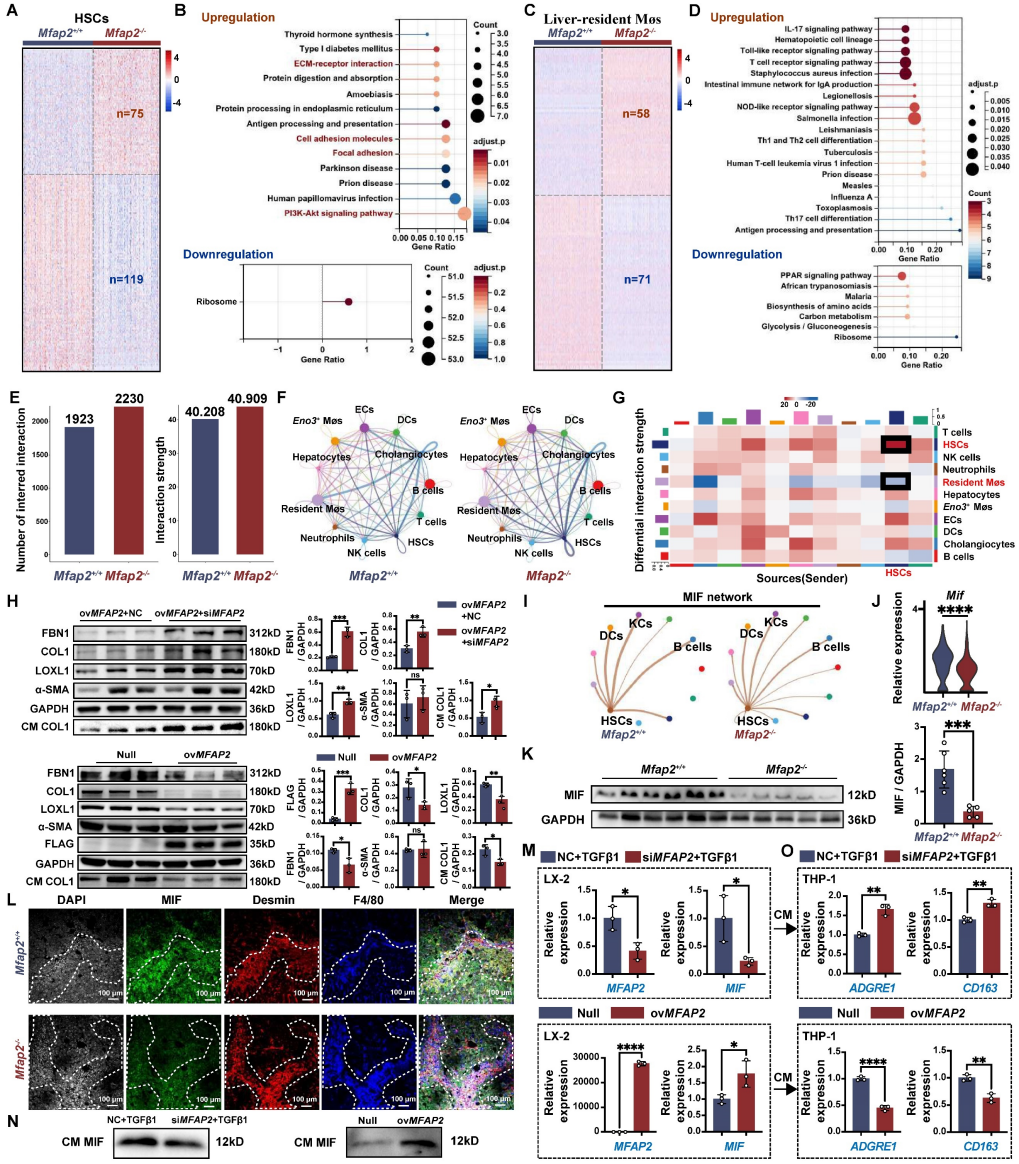
***Mfap2* deletion aggravates CCl_4_-induced liver fibrosis by stabilizing the ECM and facilitating intrahepatic inflammation.*** Mfap2*^-/-^ and *Mfap2*^+/+^ mice were injected with CCl_4_ for eight weeks. (**A, C**) Heatmaps showing the expression of differentially expressed genes in HSCs or liver-resident Møs. (**B, D**) Significantly enriched KEGG pathways based on the upregulated or downregulated genes in HSCs or liver-resident Møs (adjusted *p* < 0.05). (**E**) Number of inferred cellular interactions and their strength. (**F**) Circle plots showing the cellular interactions, with edge width proportional to the number of interactions. (**G**) Heatmap depicting differential interaction strength (decrease, blue; increase, red). The top vertical bar represents incoming signaling, while the left horizontal bar refers to outgoing signaling. (**H**) Immunoblotting analysis of FBN1, COL1, LOXL1, α-SMA, and FLAG in HSC cell lines treated with ov*Mfap2* (2.0 μg/mL, 48 hours) + NC (50 nM, 36 hours), ov*Mfap2* (2.0 μg/mL, 48 hours) + si*Mfap2* (50 nM, 36 hours), Null (2.0 μg/mL, 48 hours), or ov*Mfap2* (2.0 μg/mL, 48 hours) (n = 3/group). (**I**) Circle plots showing the interaction strength of the MIF signal from HSCs to other cells. (**J**) Violin plot depicting the expression level of *Mif* in HSCs based on scRNA-seq data. (**K**) Immunoblotting analysis of MIF protein levels (n = 5-6/group). (**L**) Multiplex IF staining of MIF (green), Desmin (red), F4/80 (blue), and DAPI (nucleus, grey). Co-localization of Desmin and MIF is indicated in yellow. (**M**) qPCR analysis of *MFAP2* and *MIF* gene expression in HSCs treated with si*MFAP2* (50 nM, 36 hours) *+* TGFβ1 (10 ng/mL, 48 hours) or ov*MFAP2* (2.0 μg/mL, 48 hours) (n = 3/group)*.* (**N**) Immunoblotting analysis of MIF levels in the CM from cell groups in (M) (n = 3/group). (**O**) qPCR analysis of *ADGRE1* and *CD163* gene expression in THP-1 cells treated with the CM from (M) for 36 hours (n = 3/group). Data are expressed as mean ± SEM. *p < 0.05, **p < 0.01, ***p < 0.001, ****p < 0.0001. ns: not significant; CM: conditioned media; NC: negative control.

To elucidate the cellular and molecular mechanisms underlying the enhanced ECM stabilization and intrahepatic inflammation due to *Mfap2* ablation, we analyzed the scRNA-seq data from NPCs isolated from the livers of *Mfap2*^-/-^ and *Mfap2*^+/+^ mice after eight weeks of CCl_4_ injection. Differential expression analysis revealed that 75 genes were significantly increased in HSCs of *Mfap2*^-/-^ mice, with these genes linked to ECM remodeling pathways, including ECM-receptor interaction, cell adhesion molecules, FA, and PI3K-Akt signaling (**Figure 6A-B**). In liver-resident Møs, the loss of *Mfap2* upregulated 58 genes, which were enriched in immune or infection-related signaling (**Figure 6C-D**). Conversely, 119 and 71 genes related to ribosome or PPAR signaling were downregulated in HSCs and liver resident Møs following *Mfap2* deletion (**Figure 6A-D**). These findings further confirm that *Mfap2* deficiency contributes to ECM stabilization and exacerbates inflammation at both the cellular and molecular levels.

Subsequently, we inferred the intercellular communication networks from the scRNA-seq data using *CellChat* 24, based on known receptor-ligand pairs. *Mfap2* ablation moderately increased both the number and the strength of intercellular signals among HSCs (**Figure 6E-G**). Specifically, the known pro-fibrotic signals SPP1 40 and TGFβ2 41 were more active among HSCs from *Mfap2*^-/-^ mice than those from *Mfap2*^+/+^ mice (**Figure S6**). Further *in vitro* studies demonstrated that inhibiting *MFAP2* in HSCs enhanced the expression of intracellular FBN1, COL1, and LOXL1, as well as COL1 secretion; in contrast, ov*MFAP2* in HSCs had the opposite effect (**Figure 6H**). Notably, neither inhibiting nor overexpressing *MFAP2* expression affected HSC activation (**Figure 6H**), which agrees with our *in vivo* findings. In conclusion, the loss of *Mfap2* stabilizes the ECM by increasing the production and secretion of matrisome proteins.

Despite increased interactions observed between HSCs and their neighboring liver-resident Møs, the strength of their crosstalk was moderately weakened following *Mfap2* ablation (**Figure 6G**). Among all the downregulated ligand-receptor signals, MIF signaling from HSCs to liver-resident Møs showed the most significant decrease (**Figure 6I** and** S6**). MIF was initially recognized as a pleiotropic cytokine arresting random Møs movement 42. Recent studies using *Mif*^-/-^ and *Mif*^Tg^ mice have demonstrated MIF's anti-fibrotic and anti-inflammatory effects in experimental liver fibrosis 43-45. Given this context, we speculate that the loss of *Mfap2* exacerbates hepatic inflammation by reducing MIF. Indeed, *Mfap2* deletion resulted in decreased MIF expression in the liver, particularly in HSCs (**Figure 6J-K**). Multiplex IF staining showed that liver peri-septal areas were enriched with MIF^+^Desmin^+^ HSCs in *Mfap2*^+/+^ mice, whereas MIF^-^ Desmin^+^ HSCs in *Mfap2*^-/-^ mice (**Figure 6L**). Meanwhile, F4/80^+^ Møs were more abundant near MIF^-^ Desmin^+^ spots in *Mfap2*^-/-^ mice (**Figure 6L**). To further gain insight into the effect of MFAP-2 on MIF production in HSCs and the recruitment of Møs, we performed *in vitro* validation experiments. As shown in **Figure 6M-N**, silencing *Mfap2* in HSCs inhibited MIF expression and secretion, while overexpression of *Mfap2* induced MIF production. We then transferred the CM from HSCs with either *MFAP2* inhibition or overexpression onto the human monocyte THP-1 cell line. As expected, the CM from HSCs with *MFAP2* inhibition increased the expression of *ADGRE1* and *CD163*, known markers of Møs, in THP-1 cells compared to the control; conversely, the CM from HSCs with *MFAP2* overexpression reduced *ADGRE1* and *CD163* gene expression in THP-1 cells (**Figure 6O**). Our results indicate that the loss of *MFAP2* in HSCs exacerbates Møs infiltration by reducing MIF levels in HSCs. Moreover, *MFAP2* inhibition in THP-1 cells, in contrast to its effect in HSCs, reduced the expression of Mø markers (**Figure S7**), highlighting that the inflammation exacerbation observed in *Mfap2*^-/-^ mice is primarily attributable to the loss of *Mfap2* in HSCs.

### OvMfap2 in HSCs protects against intrahepatic inflammation, inhibits ECM stabilization, prevents activation of FA signaling, and facilitates liver fibrosis regression

**Figure 7:**
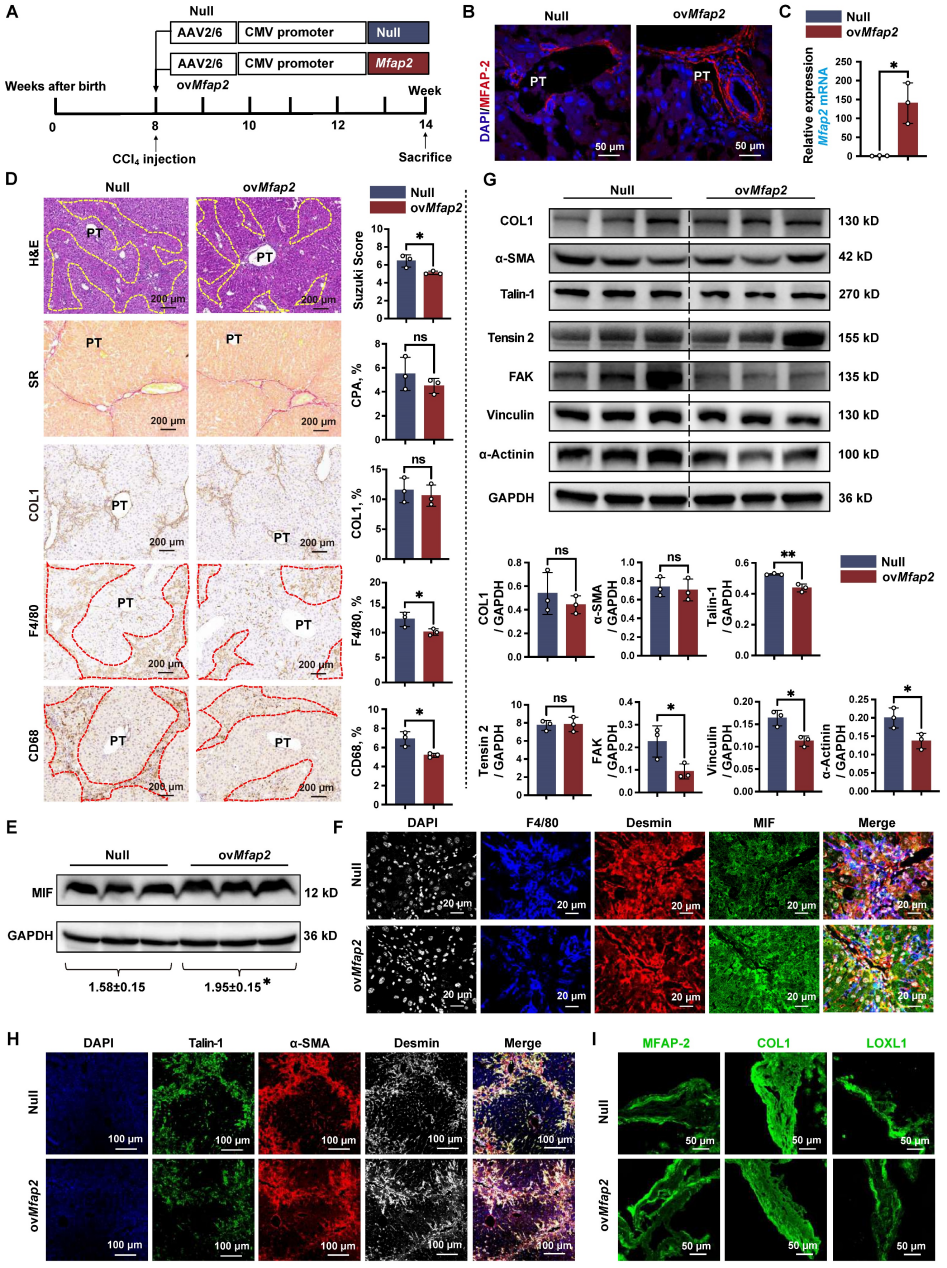
** Ov*Mfap2* prevents intrahepatic inflammation and ECM remodeling.** Ov*Mfap2* and control (Null) mice were injected with CCl_4_ for six weeks. (**A**) Schematic diagram illustrating the model of CCl_4_-induced liver fibrosis in ov*Mfap2* and Null mice. (**B**) Immunofluorescent staining of MFAP-2. (**C**) qPCR of *Mfap2* gene expression (n = 3/group). (**D**) H&E staining, Sirius Red staining, IHC analysis of COL1, F4/80, and CD68 (n = 3/group). (**E**) Immunoblotting analysis of MIF levels after ov*MFAP2* in LX-2 cells (n = 3/group). (**F**) Multiplex IF staining of MIF (Green), Desmin (red), F4/80 (blue), and DAPI (nucleus, grey). Co-localization of MIF and Desmin is shown in yellow. (**G**) Immunoblotting analysis of COL1, α-SMA, FA signaling markers after ov*MFAP2* in LX-2 cells (n = 3/group). (**H**) Multiplex IF staining of Talin-1 (Green), α-SMA (red), Desmin (grey), and DAPI (nucleus, blue). Co-localization of Talin-1 and α-SMA is shown in yellow. (**I**) Immunofluorescent staining of COL1 (green) or LOXL1 (green) in decellularized ECM scaffolds. Data are expressed as mean ± SEM. *p < 0.05, **p < 0.01. ns: not significant.

To further elucidate the protective role of HSC-enriched MFAP-2 in chronic CCl_4_ injury, we administrated mice with AAV6-*Mfap2* vector via the tail vein injection in mice, followed by CCl_4_ treatment for six weeks (**Figure 7A**). We confirmed the overexpression of *Mfap2* through immunofluorescence, qPCR, and immunoblotting (**Figure 7B-C** and** S8A**). Consistent with the findings from *Mfap2* knockout studies, ov*Mfap2* did not significantly alter the liver-to-body weight ratio, serum ALT and AST levels (**Table S3**), ECM accumulation, or the expression levels of COL1 and α-SMA in the liver (**Figure 7D**). These results indicate that ov*Mfap2* in HSCs does not affect the histological progression of liver fibrosis.

However, ov*Mfap2* decreased the Suzuki score, *Tnf* expression, and the number of F4/80^+^ or CD68^+^ Møs adjacent to lobular areas, contrasting with our observations following *Mfap2* deletion (**Figure 7D**, **S4B** and** S5B**). Moreover, immunoblotting and multiplex IF confirmed that ov*Mfap2* increased MIF expression in Desmin^+^ HSCs while reducing peri-septal infiltration of F4/80^+^ Møs near HSCs (**Figure 7E-F**). Additionally, ov*Mfap2* downregulated key FA signaling components (Talin-1, FAK, Vinculin, α-Actinin) (**Figure 7G**), indicating a suppression of FA signaling. Subsequent multiplex IF analysis confirmed that ov*Mfap2*-induced FA signaling suppression mainly occurred in HSCs, as evidenced by decreased Talin-1 expression in α-SMA^+^Desmin^+^ HSCs (**Figure 7H**). Furthermore, ov*Mfap2* increased MFAP-2 expression while reducing the levels of COL1 and LOXL1 in the decellularized ECM scaffolds (**Figure 7I**). This finding contrasts with the observations following *Mfap2* depletion, suggesting a reduced ECM stabilization after ov*Mfap2* in HSCs.

Next, we examined the effects of *Mfap2* overexpression in HSCs on liver fibrosis regression. Both Null and ov*Mfap2* mice, which underwent six weeks of CCl_4_ injection, were allowed to recover (**Figure S9A-B**). Following a three-week recovery after CCl_4_ cessation, the liver-to-body weight ratio, serum ALT level, and intrahepatic inflammation were comparable between Null and ov*Mfap2* mice (**Table S3** and **Figure S9C**). However, immunostaining and morphometric analysis of COL1 indicated that ov*Mfap2* mice displayed less pronounced, thinner, and discontinuous collagen fibers in the liver septal regions compared to Null mice (**Figure S9D**). Additionally, ov*Mfap2* mice showed suppressed activity of liver FA signaling compared to Null mice after three weeks of fibrosis regression (**Figure S9E**). These results indicate a more rapid regression of fibrosis in the ov*Mfap2* group. In agreement with the findings from mouse studies, we also observed that patients with significant liver fibrosis and higher baseline *MFAP2* expression were more likely to experience fibrosis regression after etiology control (**Figure S10**).

### MFAP-2 exerts a more pronounced effect on liver fibrosis in BDL mouse models

**Figure 8:**
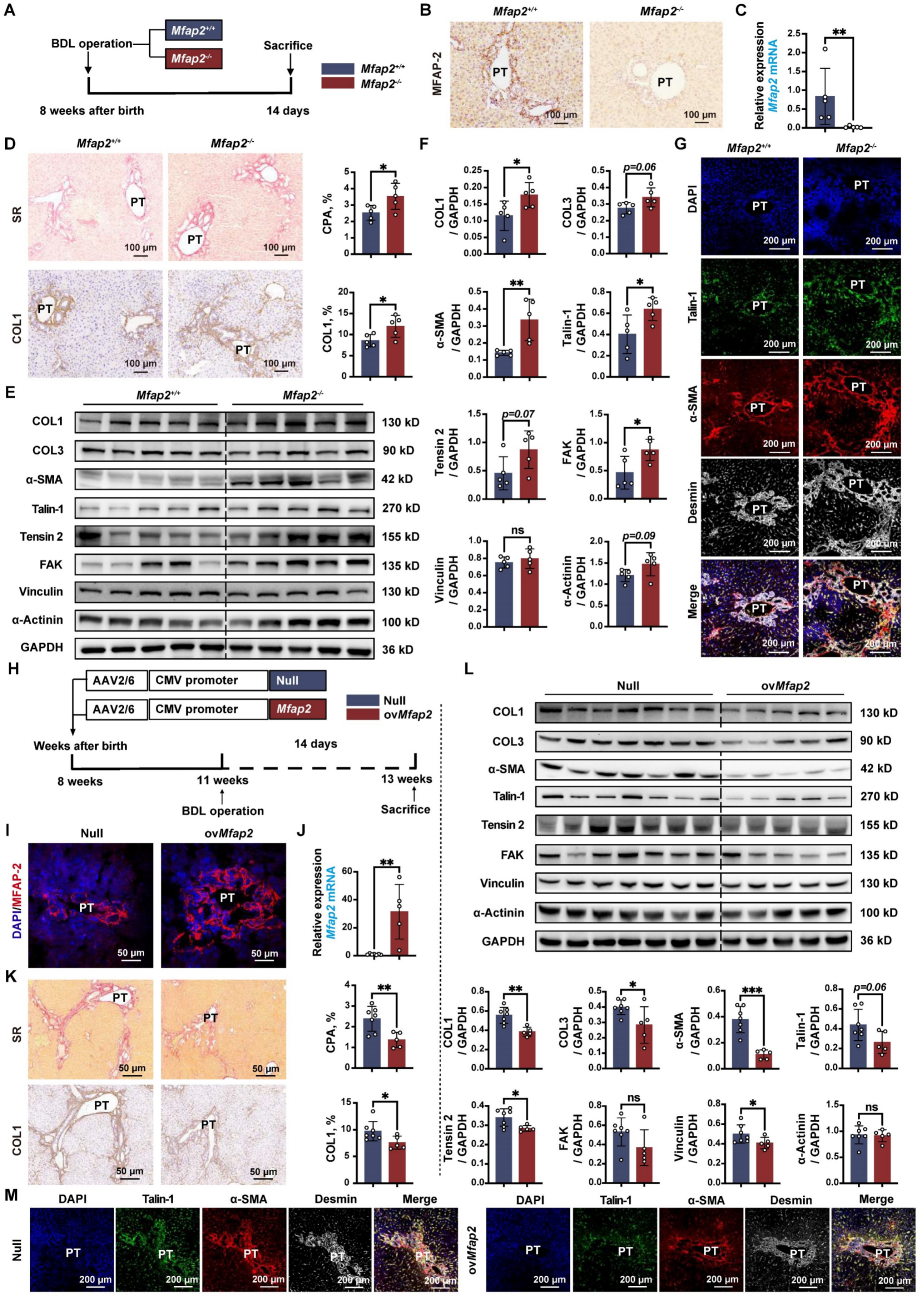
***Mfap2*^-/-^ aggravates, while ov*Mfap2* attenuates liver fibrosis in the BDL mouse model.** *Mfap2***^-/-^**,* Mfap2*^+/+^, Null, or ov*Mfap2* mice underwent BDL for fourteen days. (**A**) Schematic diagram illustrating BDL-induced liver fibrosis in *Mfap2*^+/+^ and *Mfap2*^-/-^ mice. (**B, C**) IHC and qPCR analyses of MFAP-2 (*Mfap2*) (n = 5/group). (**D**) Sirius Red staining and IHC analysis of COL1 (n = 5/group). (**E, F**) Immunoblotting analysis of COL1, COL3, α-SMA, and FA signaling markers (n = 5/group). (**G**) Multiplex IF staining of Talin-1 (green), α-SMA (red), Desmin (grey), and DAPI (nucleus, blue). Co-localization of Talin-1 and α-SMA is indicated in yellow. (**H**) Schematic diagram illustrating BDL-induced liver fibrosis in Null and ov*Mfap2* mice. (**I, J**) Immunofluorescent staining of MFAP-2 (red) and qPCR analysis of *Mfap2* (n = 5-7/group). (**K**) Sirius Red staining and IHC analysis of COL1 (n = 5-7/group). (**L**) Immunoblotting analysis of COL1, COL3, α-SMA, and FA signaling markers (n = 5-7/group). (**M**) Multiple IF staining of Talin-1 (green), α-SMA (red), Desmin (grey), and DAPI (nucleus, blue). Co-localization of Talin-1 and α-SMA is shown in yellow. Data are expressed as mean ± SEM. *p < 0.05, **p < 0.01, ***p < 0.001. ns: not significant.

Lastly, we validated the role of MFAP-2 using the well-established BDL model of portal fibrosis. *Mfap2* ablation resulted in enhanced ECM deposition and increased expression of COL1, α-SMA, and FA signaling proteins (Talin-1, FAK) compared to *Mfap2*^+/+^ mice, fourteen days post-BDL (**Figure 8A-F**). The activation of FA signaling following *Mfap2* ablation was observed specifically in α-SMA^+^Desmin^+^ HSCs, as illustrated in **Figure 8G**. In contrast, ov*Mfap2* via an AAV6-*Mfap2* vector (**Figure 8H-J** and **S8B**) delayed ECM accumulation and reduced markers of fibrosis (COL1, α-SMA) as well as FA signaling (Tensin-2, Vinculin) (**Figure 8K-L**). Additionally, ov*Mfap2* decreased the number of Talin-1^+^α-SMA^+^ HSCs after BDL (**Figure 8M**). These findings suggest that MFAP-2 exerts a more pronounced effect on liver fibrosis in the BDL model compared to the CCl_4_ model of liver fibrosis.

However, neither *Mfap2*^-/-^ nor ov*Mfap2* in the BDL model affected liver inflammation, as evidenced by similar levels of pro-inflammatory genes, Suzuki scores, the number of infiltrating F4/80^+^ or CD68^+^ Møs, as well as serum ALT and AST levels (**Figure S4C-D**,** S5C-D, S11** and **Table S3**). These findings contrast with previous observations in the CCl_4_ model. We observed that MIF was primarily expressed in hepatocytes and remained unchanged in the livers of both *Mfap2*^-/-^ and ov*Mfap2* mice compared to their respective controls, fourteen days post-BDL (**Figure S12**). In summary, *Mfap2* ablation exacerbates liver fibrosis, while ov*Mfap2* in HSCs mitigates fibrosis in the BDL model without impacting intrahepatic inflammation.

## Discussion

Our current study found that the structure of elastin-rich tissues in *Mfap2*^-/-^ mice remained unchanged (**Figure S13**), mirroring an earlier study 9, thereby challenging the notion of MFAP-2's indispensableness in ECM assembly. *Mfap2*^-/-^ or ov*Mfap2* mice did not develop spontaneous pathological abnormalities in the liver in the absence of intoxication stimulation (**Figure S14**), likely due to the limited presence of MFAP-2 in normal livers. Similar to findings in adipose tissue and dermis 34, 35, our study revealed that MFAP-2 is predominantly expressed in activated HSCs. Unexpectedly, under prolonged chemical injury, the absence of *Mfap2* did not result in significant changes in overall collagen deposition, which contrasts with a recent report by Sun et al. that used the lentivirus delivery system targeting *Mfap2* 11. However, we observed that *Mfap2* ablation contributed to ECM stabilization and exacerbated intrahepatic inflammation. In contrast, ov*Mfap2* in HSCs using an AAV6 vector, known for its myofibroblast tropism 13, 46, reduced ECM stabilization and intrahepatic inflammation resulting from chronic CCl_4_ intoxication. Moreover, we found that *Mfap2* ablation slowed spontaneous regression of fibrosis, while *Mfap2* overexpression promoted it upon CCl_4_ cessation. Additionally, treatment-naïve patients with significant liver fibrosis and higher levels of liver *MFAP2* gene expression tend to experience fibrosis regression following etiology control. These findings underscore the role of HSC-enriched MFAP-2 in liver fibrosis regression.

Our proteomic analysis of ECM scaffolds revealed that *Mfap2* ablation significantly increased while ov*Mfap2* decreased insoluble collagens and LOXL1 latent in decellularized ECM scaffolds following chronic CCl_4_ injury. LOXL1 is known to catalyze the cross-linking of the fibrotic matrix 38, 39, resulting in ECM stabilization and increased resistance to proteolytic degradation 47. Therefore, *MFAP2* deletion or overexpression likely influences the reversibility of liver fibrosis by regulating the stabilization of the fibrotic matrix. A prior study indicated that intracellular MFAP-2 transcriptionally inhibits the expression of genes encoding ECM components 48. Supporting this, our scRNA-seq analysis demonstrated an upregulation of ECM-related genes in HSCs from *Mfap2*^-/-^ mice after eight weeks of CCl_4_ injection. In our *in vitro* studies, we showed that HSC-enriched MFAP-2 antagonizes the production and secretion of matrisome proteins, including COL1, FBN1, and LOXL1. This effect is independent of HSC activation, as changes in *MFAP2* levels did not affect α-SMA expression. Additionally, we observed that MFAP-2 can influence FA signaling, which usually precedes HSC activation 49. The activation of FA signaling following *Mfap2* ablation suggests ECM stabilization and inhibition of collagen degradation through a decrease in matrix metalloproteinases 50. This represents a positive feedback loop that stiffens the ECM in the setting of *Mfap2* deletion.

Moreover, we observed that *Mfap2* deficiency increased the recruitment of Møs to the peri-septal areas in the CCl_4_ model. The exacerbated intrahepatic inflammation resulting from *Mfap2* deletion likely contributed to the delayed regression of fibrosis. Previous studies have indicated that MFAP-2 has anti-inflammatory properties by suppressing TGFβ1 in both cellular and matrix milieus 7-10; however, this does not appear to hold true in the liver. In this study, we found both cellular active and latent TGFβ1 levels in the ECM were unaffected by *Mfap2* deletion or overexpression (**Figure S15**). In fact, during chronic CCl_4_ injury, *Mfap2* deletion reduced MIF in HSCs, while F4/80^+^ Møs were more abundant near MIF^-^Desmin^+^ spots in the liver, suggesting that MFAP-2 orchestrates intrahepatic inflammation by regulating MIF in HSCs. Our *in vitro* studies further confirmed the positive effect of MFAP-2 on MIF production and Mø activation. Notably, MIF was originally recognized as a pleiotropic cytokine responsible for inhibiting Mø migration 42 and has recently been shown to possess anti-fibrotic and anti-inflammatory properties in experimental liver fibrosis using gene-edited mice 43-45. Additionally, a recent study found that *MFAP2*^+^ cancer-associated fibroblasts communicate with Møs by secreting MIF, which exerts immunosuppressive effects 51. The mechanisms by which intracellular MFAP-2 regulates MIF production in HSCs require further investigation.

In addition to CCl_4_ models, we also validated the role of MFAP-2 in the BDL model. We observed that *Mfap2^-/-^* mice developed severe liver fibrosis, as evidenced by a significant increase in collagen deposition following BDL. In contrast, ov*Mfap2* mice exhibited impeded fibrosis progression under the same conditions. However, neither *Mfap2*^-/-^ nor ov*Mfap2* affected intrahepatic inflammation in BDL mice, which differs from the findings in the CCl_4_ model. In the CCl_4_ model, *Mfap2* deletion moderately affected collagen deposition, likely due to the recruitment of peri-lobular Møs, which are known to secrete matrix metalloproteinases that degrade the ECM 52. A recent study revealed that elevated bile acid levels stemming from cholestasis impair the functionality of Møs 53, potentially explaining the divergent effects of MFAP-2 on Møs in CCl_4_ and BDL-induced liver fibrosis. Additionally, we noted that MIF was expressed in hepatocytes rather than HSCs, and its expression remained unchanged by *Mfap2*^-/-^ or ov*Mfap2* post-BDL. Notably, liver MIF levels significantly increased in the BDL model but decreased in the CCl_4_ model (**Figure S16**). These findings also elucidate the different effects of *Mfap2*^-/-^ and ov*Mfap2* on inflammation between the CCl_4_ and BDL models. Furthermore, Muynck et al. recently reported that CD11b^+^F4/80^+^TIM4^+^ Møs were reduced in mice with BDL compared to sham controls 54, likely due to a significant upregulation of MIF, which merits further investigation.

Certainly, our study has limitations. In fibrotic mice, the combination of immunofluorescent co-localization experiments and scRNA-seq analysis indicated that *Mfap2* is predominantly enriched and upregulated in activated HSCs, with minimal expression observed in other liver cell types. However, in normal mice, analysis of primary liver cells showed that *Mfap2* is not exclusively expressed in quiescent HSCs, but with similar and minimal levels in hepatocytes and Møs (**Figure S17**). Therefore, the *Mfap2*^-/-^ mouse serves as an ideal gene-edited model to investigate the role of HSC-enriched MFAP-2 in liver fibrosis, rather than in normal physiological conditions. In contrast, we employed the AAV6-*Mfap2* vector to generate a mouse model with HSC-overexpressed MFAP-2. While the AAV6 vector has demonstrated myofibroblast tropism 13, 46, and our quality control experiments confirmed successful MFAP-2 overexpression in HSCs, AAV6 also showed organ tropism for skeletal muscle, heart, and spleen 46. Therefore, the development of more robust *in vivo* tools, such as HSC-specific *Mfap2* knockout or knock-in mice generated via the Cre/loxP system, is essential. Moreover, although our findings underscore the pivotal role of HSC-derived MFAP-2 in regulating ECM stabilization and inflammation by enhancing matrisome composition and reducing MIF production, the precise molecular mechanisms by which intracellular MFAP-2 influences the matrisome and MIF in HSCs warrant further investigation. Additionally, although MFAP-2 exhibits a protective effect against fibrosis, its impact may largely depend on the localization and dysregulation patterns of MIF, which can vary across different etiologies. This variability may influence the broader applicability of MFAP-2-based therapeutic efficacy and warrants further investigation.

In conclusion, MFAP-2 levels increase compensatorily in fibrotic livers, primarily within activated HSCs. In the CCl_4_ model, deletion of *Mfap2* stabilizes the ECM by promoting the production and secretion of matrisome proteins, while simultaneously exacerbating intrahepatic inflammation through producing and secreting MIF. In the BDL model, *Mfap2* deletion leads to a more pronounced pro-fibrotic effect in the absence of inflammation. Moreover, ov*MFAP2* may protect against liver fibrosis and promote its regression, potentially offering significant clinical benefits for patients whose fibrosis does not regress after etiology removal. For future clinical applications, the AAV6 vector, noted for its enhanced safety profile, reduced immunogenicity, and superior long-term efficacy in regulating gene expression 13, 55-57, emerges as a promising candidate for MFAP2-based therapy for liver fibrosis. In addition to AAV6, the use of liposomes and other innovative nanomaterial-based targeted delivery systems may serve as alternative vectors for delivering *MFAP2* to activated HSCs in the treatment of liver fibrosis.

## Supplementary Material

Supplementary figures and tables.

## Abbreviations

AAV: adeno-associated virus
BDL: bile duct ligation
CCl_4_: carbon tetrachloride
CM: conditioned media
ECM: extracellular matrix
FA: focal adhesion
HSC: hepatic stellate cell
IF: immunofluorescence
KEGG: Kyoto Encyclopedia of Genes and Genomes
LC-MS/MS: label-free liquid chromatography-tandem mass spectrometry
Mø: macrophage
MIF: macrophage migration inhibitory factor
MFAP-2: microfibrillar-associated protein 2
NPC: non-parenchymal cell
scRNA-seq: single-cell RNA sequencing
SDS: sodium dodecyl sulfate
TGF: transforming growth factor
