# Supplementary material for: Hepatic stellate cell-derived microfibrillar-associated protein 2 prevents liver fibrosis by regulating extracellular matrix and inflammation
Source: Theranostics. 2025 Mar 10;15(9):4033–53. doi: 10.7150/thno.109771 (PMC11980660; doi:10.7150/thno.109771)
Supplement: Supplementary file 1 — Supplementary figures and tables. [file thnov15p4033s1.pdf]

Supplementary materials for

Hepatic stellate cell-derived microfibrillar-associated protein 2 prevents liver fibrosis by regulating extracellular matrix and inflammation

Wen Zhang[1,2,3,#], Wenyue Wu[1,2,3,#], Ning Zhang[1,2,3], Hong Li[2,3,4,5], Yameng Sun[1,2,3,6], Xiaodong Ge[7], Hui Han[7], Shuyan Chen[1,2,3], Anjian Xu[2,3,4,5], Sai Santosh Babu Komakula[7], Chao Wang[7], Nithyananthan Subramaniyam[7], Qi Han[1,2,3], Aiting Yang[2,3,4,5], Xuzhen Yan[2,3,4,5], Natalia Nieto[7,8,9,*], Hong You[1,2,3,9,*], Wei Chen[2,3,4,5,6,7,*]

[1] Liver Research Center, Beijing Friendship Hospital, Capital Medical University, Beijing 100050, China.

[2] State Key Lab of Digestive Health, Beijing Friendship Hospital, Capital Medical University, Beijing 100050, China.

[3] National Clinical Research Center of Digestive Diseases, Beijing Friendship Hospital, Capital Medical University, Beijing 100050, China.

[4] Experimental and Translational Research Center, Beijing Friendship Hospital, Capital Medical University, Beijing 100050, China.

[5] Beijing Clinical Research Institute, Beijing Friendship Hospital, Capital Medical University, Beijing 100050, China.

[6] Chinese Institutes for Medical Research (CIMR), Beijing 10069, China.

[7] Department of Pathology, University of Illinois Chicago, Chicago, IL 60612, USA.

[8] Division of Gastroenterology and Hepatology, Department of Medicine, University of Illinois Chicago, Chicago, IL 60612, USA.

[9] Authors share co-senior authorship.

[#]These authors contributed equally.

[*]**Corresponding Authors:**

Wei Chen, Experimental and Translational Research Center, Beijing Friendship Hospital, Capital Medical University, No. 95 Yong'an Road, Xicheng District, Beijing 100050, China. E-mail: cw_2011@126.com. Tel: +86 (010) 6313-9311

Hong You, Liver Research Center, Beijing Friendship Hospital, Capital Medical University, No. 95 Yong'an Road, Xicheng District, Beijing 100050, China. E-mail: youhong30@sina.com. Tel: +86 (010) 6313-9019

Natalia Nieto, Division of Gastroenterology and Hepatology, Department of Medicine, University of Illinois Chicago, 840 S. Wood St., Suite 130 CSN, MC 847, Chicago, IL 60612, USA. E-mail: nnieto@uic.edu. Tel: +1 (312) 996-7316

Running title: MFAP-2 alleviates liver fibrosis.

1. **Supplementary figures**
2. **Supplementary tables**

Supplementary figures

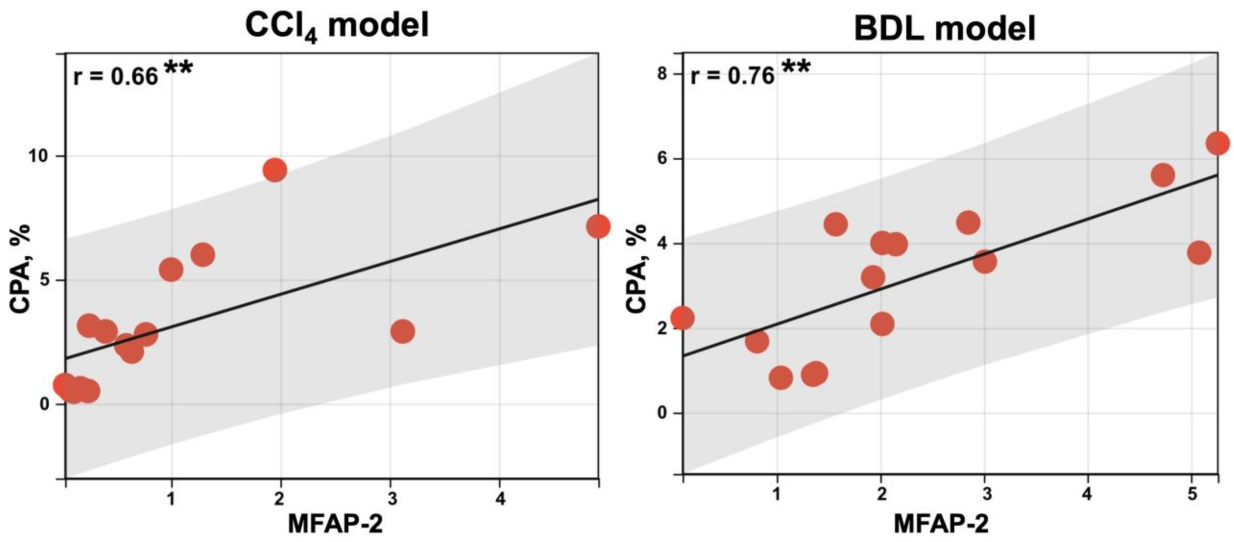

Figure S1. Correlation of liver MFAP-2 protein expression with fibrogenesis. The Pearson correlation analysis between MFAP-2 protein expression and CPA in the CCl_4 and BDL mouse models. **$p < 0.01$. CPA, collagen proportional area.

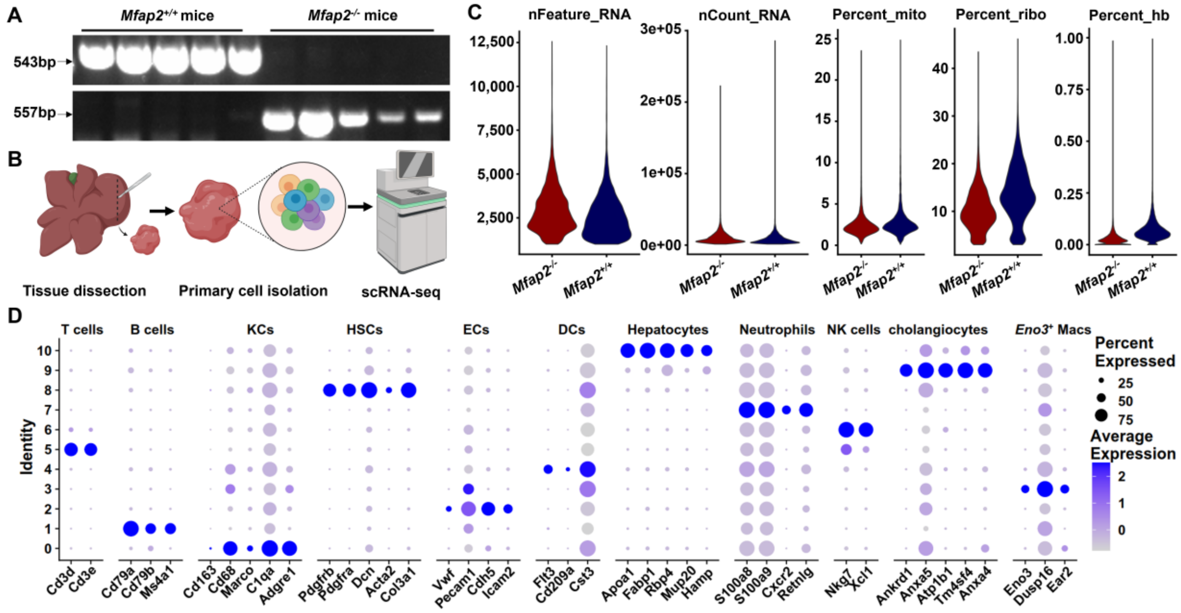

Figure S2. scRNA-seq workflow and data preprocessing. (**A**) Genotyping results show bands for *Mfap2*$^{+/+}$ alleles (543 bp) and *Mfap2*$^{-/-}$ alleles (557 bp). (**B**) Schematic representation of the cell preparation and scRNA-seq workflow. (**C**) Violin plots displaying the distribution of gene counts (nFeature_RNA), UMIs (nCount_RNA), and the

percentages of mitochondrial (percent_mito), ribosomal (percent_ribo), and hemoglobin (percent_hb) genes in NPCs isolated from *Mfap2*$^{+/+}$ and *Mfap2*$^{-/-}$ mice at eight weeks of CCl_4 injection. (**D**) Dot plot depicting the expression of marker genes for cell type annotation.

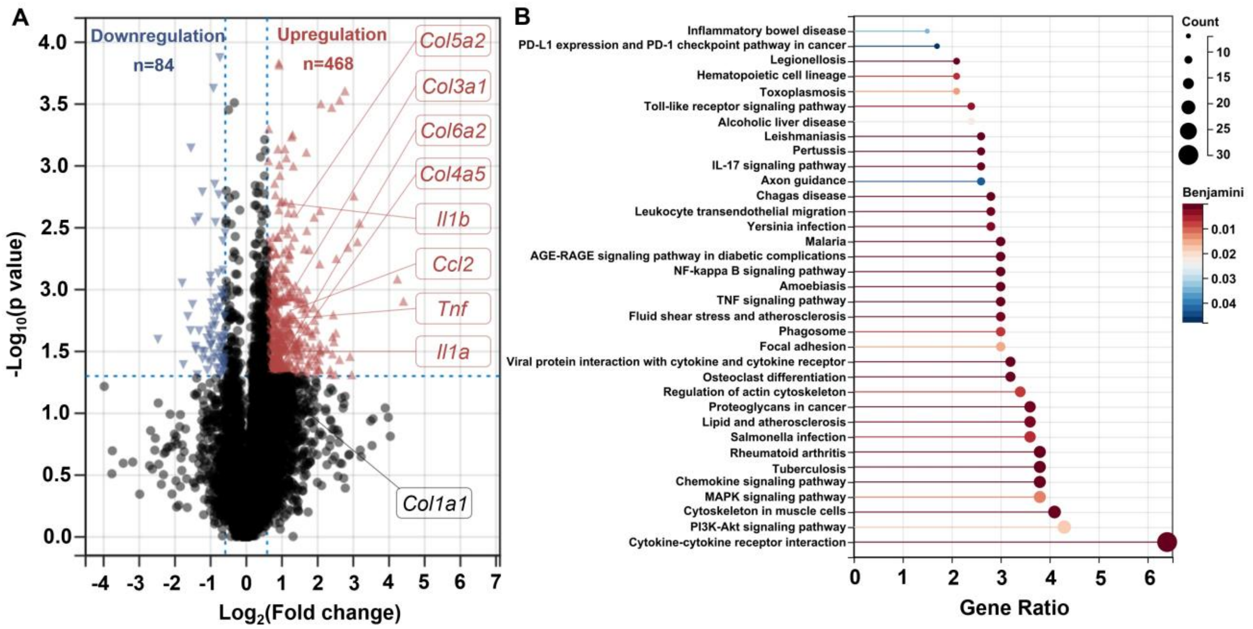

Figure S3. Increased intrahepatic inflammation and activated FA signaling in the livers from *Mfap2*$^{-/-}$ mice following CCl_4 withdrawal. Bulk RNA-seq was conducted on the livers of *Mfap2*$^{-/-}$ and *Mfap2*$^{+/+}$ mice that were administered CCl_4 for eight weeks, followed by a four-week cessation period. (**A**) The volcano plot displays differentially expressed genes (n = 4 per group; $p < 0.05$ & fold change > 1.5). (B) Significantly enriched KEGG pathways derived from upregulated genes (Benjamini-corrected $p < 0.05$).

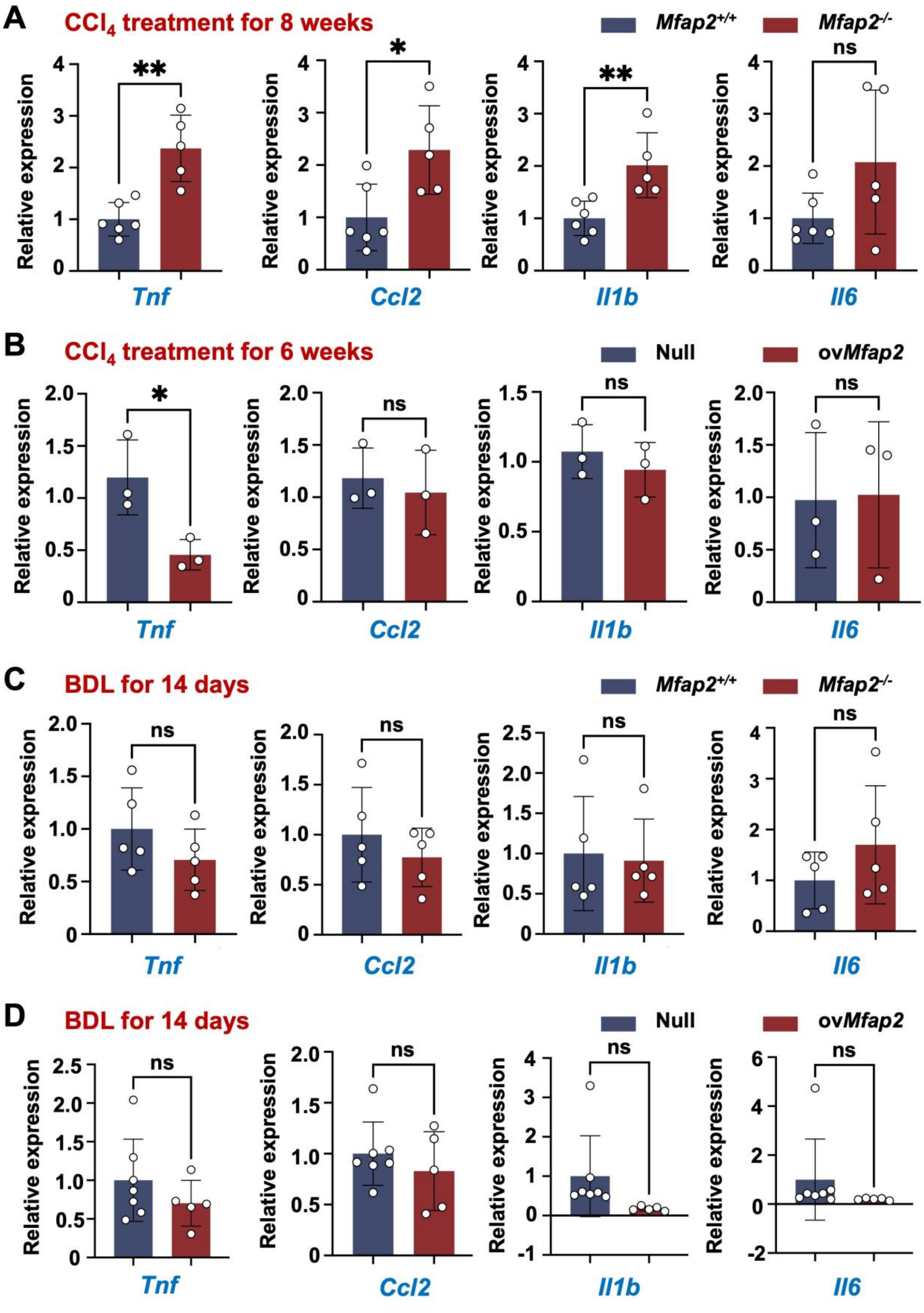
A
CCl4 treatment for 8 weeks
Mfap2+/+
Mfap2-/-
Relative expression
Tnf
Ccl2
Il1b
Il6
**
*
ns
B
CCl4 treatment for 6 weeks
Null
ovMfap2
C
BDL for 14 days
D
BDL for 14 days

Figure S4. qPCR analysis of pro-inflammatory gene expression. Gene expression of liver *Tnf*, *Ccl2*, *Il1b*, or *Il6* was compared between (**A**) *Mfap2*$^{-/-}$ and *Mfap2*$^{+/+}$ mice at eight weeks of CCl_4 injection (n = 5-6/group), (**B**) Null and ov*Mfap2* mice at six weeks of CCl_4 injection (n = 3/group), (**C**) *Mfap2*$^{-/-}$ and *Mfap2*$^{+/+}$ mice after BDL operation (n = 5/group), or (**D**) Null and ov*Mfap2* mice after BDL operation (n = 5-7/group). Data are expressed as mean ± SEM. *$p < 0.05$; **$p < 0.01$; ns, not significant.

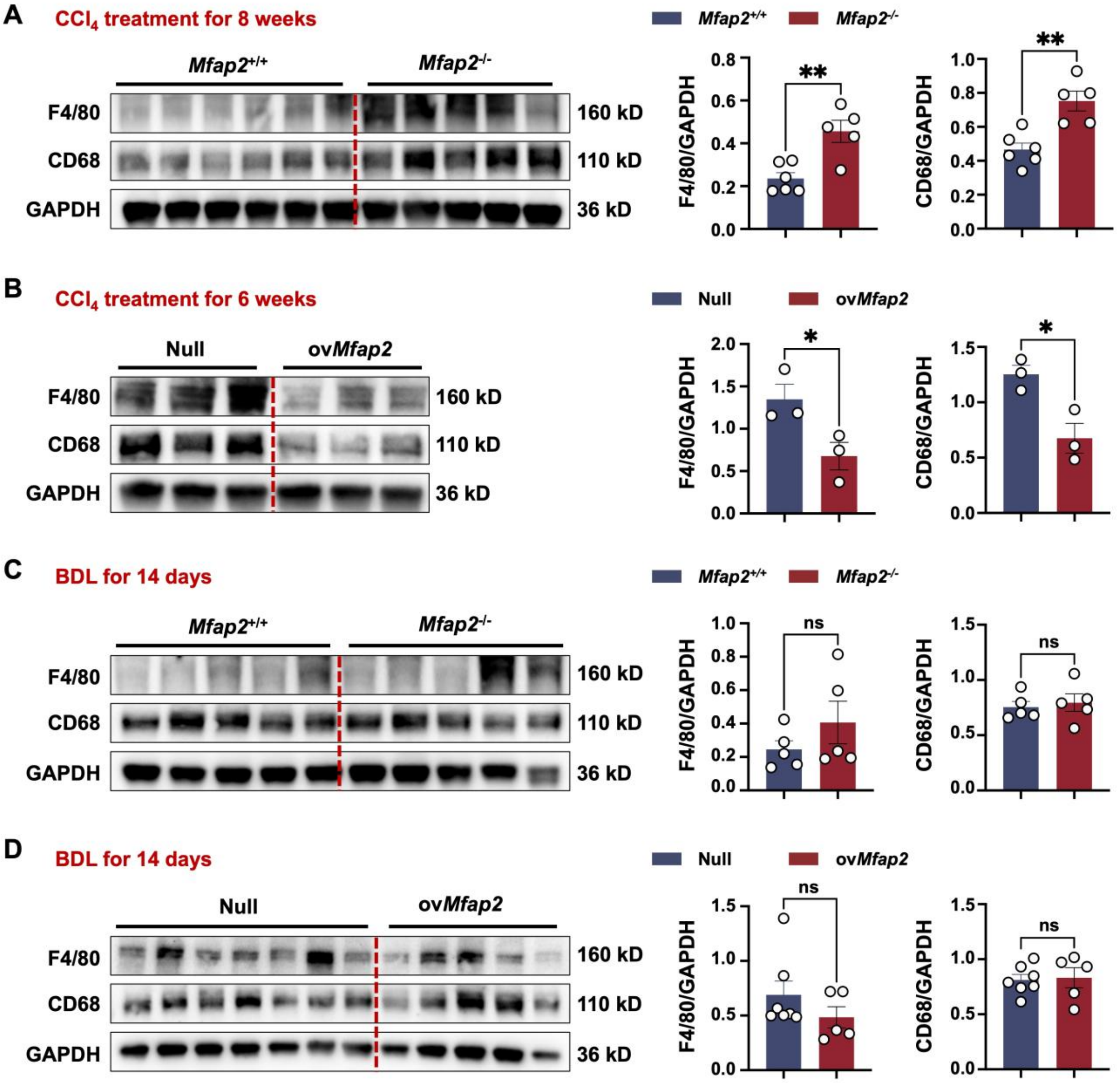

Figure S5. MFAP-2 mitigates liver inflammation in CCl_4 but not BDL mouse liver fibrosis models. Immunoblotting analysis was conducted to compare the expression of

F4/80 and CD68 proteins in liver tissues from (**A**) *Mfap2*$^{+/+}$ and *Mfap2*$^{-/-}$ mice subjected to 8 weeks of CCl_4 treatment (n = 5-6/group), (**B**) Null and ov*Mfap2* mice after 6 weeks of CCl_4 treatment (n = 3/group), and (**C**) *Mfap2*$^{+/+}$ and *Mfap2*$^{-/-}$ mice following BDL (n = 5/group), as well as (**D**) Null and ov*Mfap2* mice post-BDL (n = 5-7/group). Data are presented as mean ± SEM. *$p < 0.05$, **$p < 0.01$; ns, not significant.

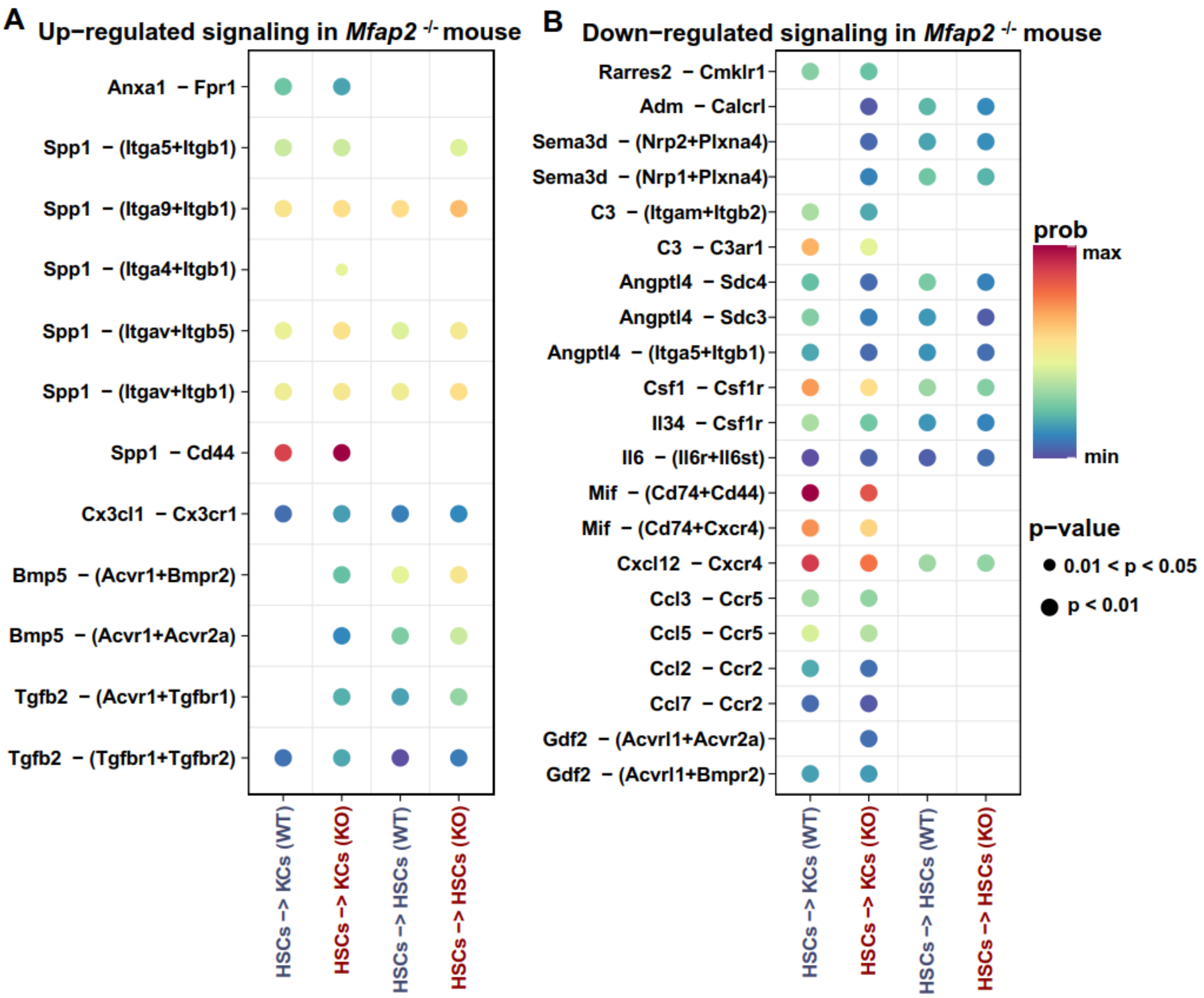

Figure S6. Alterations in signaling dynamics from HSCs to HSCs or liver-resident Møs (Kupffer cells, KCs) following *Mfap2* ablation in CCl_4-injected mice. Upregulated and downregulated signaling pathways are illustrated by comparing the communication probabilities mediated by ligand-receptor pairs from HSCs to HSCs or KCs in *Mfap2*$^{-/-}$ compared to *Mfap2*$^{+/+}$ mice at eight weeks of CCl_4 injection. The dot color

and size represent the calculated communication probability and the statistical significance, respectively.

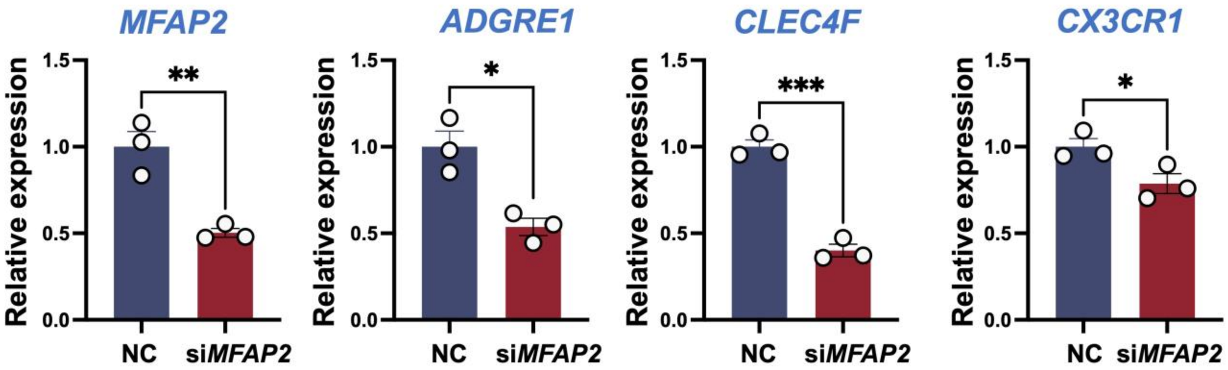

Figure S7. Silencing of *MFAP2* inhibits the activation of Møs. qPCR analysis of *MFAP2*, *ADGRE1*, *CLEC4F*, or *CX3CR1* gene expression in THP-1 cells with si*MFAP2* treatment (n = 3/group, 50 nM, 24 hours). Data are expressed as mean ± SEM. **p* < 0.05, ***p* < 0.01, ****p* < 0.001. NC, negative control.

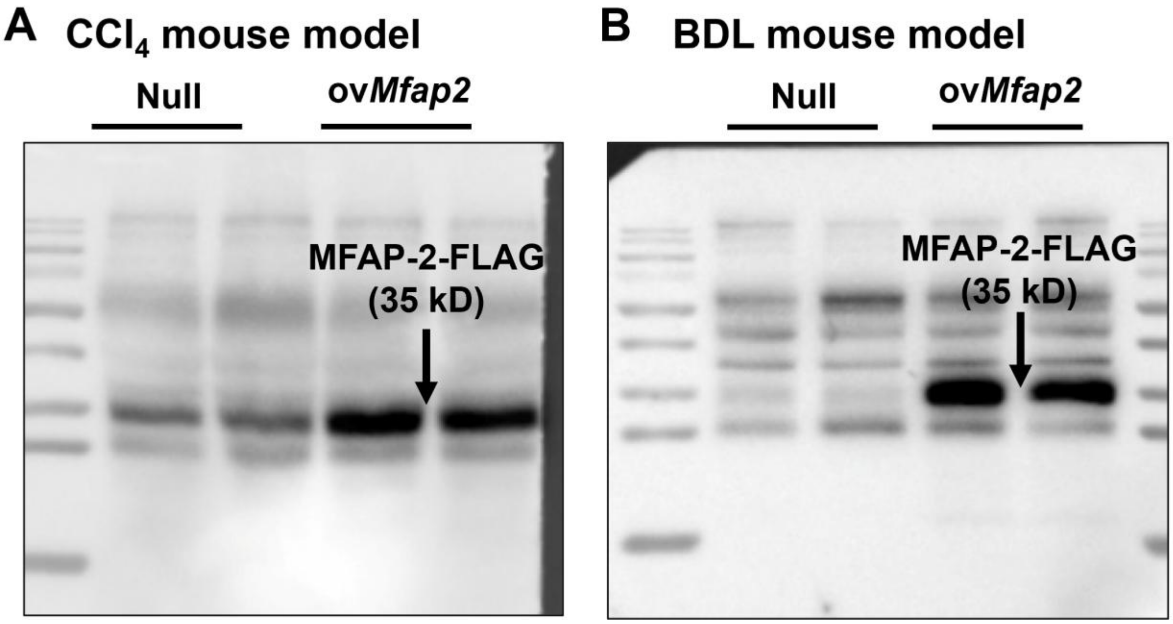

Figure S8. Verification of ov*Mfap2* expression efficiency in mice. Representative bands of immunoblotting analysis of liver FLAG expression in ov*Mfap2* mice injected intravenously with AAV6-CMV-*Mfap2*-HA-EF1a-mNeonGreen-3×FLAG-WPRE vector

and their controls (AAV6-CMV-MCS-EF1a-mNeonGreen-3×FLAG-WPRE vector, Null) in both the (**A**) CCl_4 and (**B**) BDL mouse models (n = 2/group).

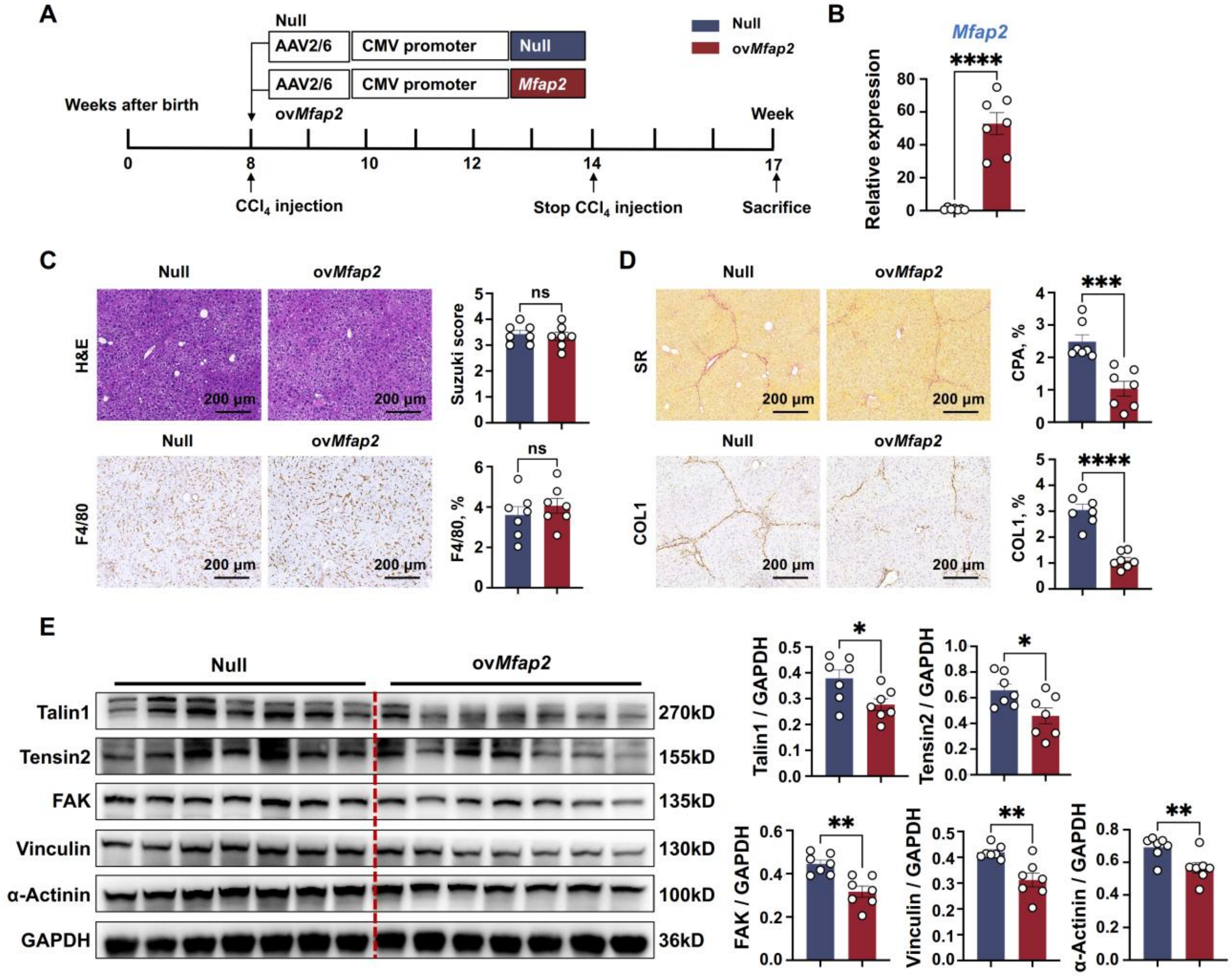

Figure S9. Ov*Mfap2* in HSCs facilitates liver fibrosis regression after CCl_4 cessation for three weeks. (**A**) Schematic diagram illustrating the model of CCl_4-induced liver fibrosis and subsequent resolution. (**B**) qPCR analysis of *Mfap2* gene expression (n = 7/group). (**C**) H&E staining of liver slices and IHC analysis of F4/80 expression (n = 7/group). (**D**) Sirius Red staining of liver sections and IHC analysis of COL1 expression (n = 7/group). (**E**) Immunoblotting analysis of FA signaling markers (n = 7/group). Data are expressed as mean ± SEM. *$p < 0.05$, **$p < 0.01$, ***$p < 0.001$, ****$p < 0.0001$.

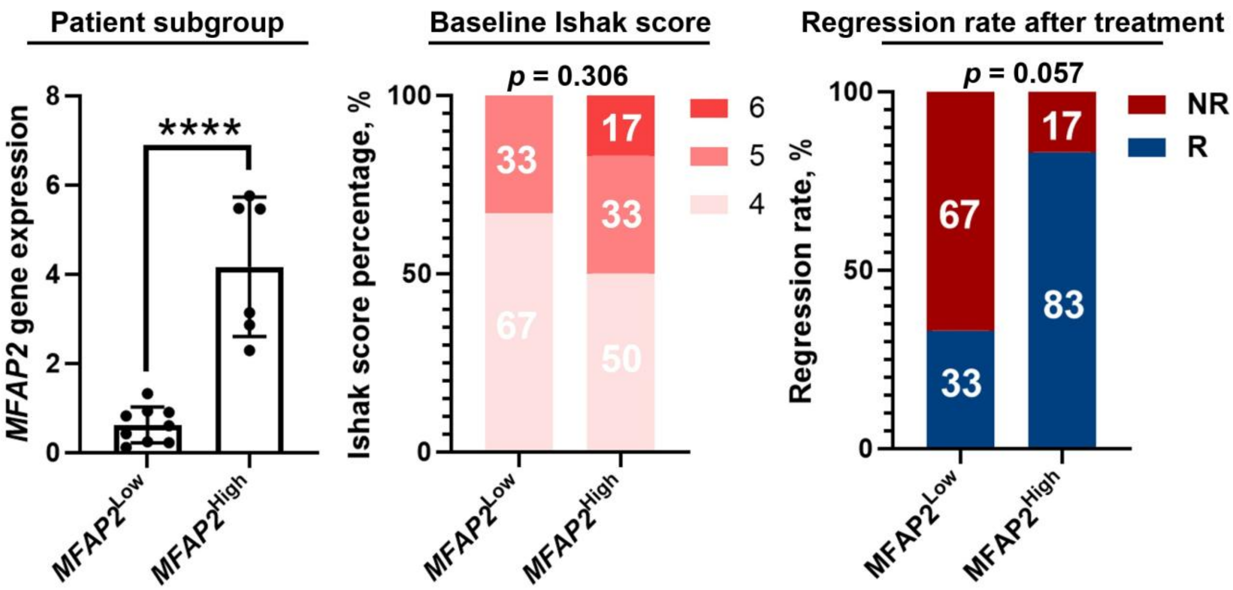

Figure S10. Patients with significant liver fibrosis and elevated baseline *MFAP2* expression are more likely to experience fibrosis regression following etiology control. A total of 15 liver transcriptomic profiles from patients with a baseline Ishak score of ≥ 4 were included in the analysis. Patients were divided into two subgroups based on baseline *MFAP2* gene expression levels: *MFAP2*^{Low} (FPKM < 2, n = 9) and *MFAP2*^{High} (FPKM > 2, n = 6). The baseline Ishak score and the rate of regression were compared between the two patient groups using Chi-square trend test and Chi-square test. Continuous data are presented as mean ± SEM, while categorical data are expressed as percentages. ****$p < 0.0001$.

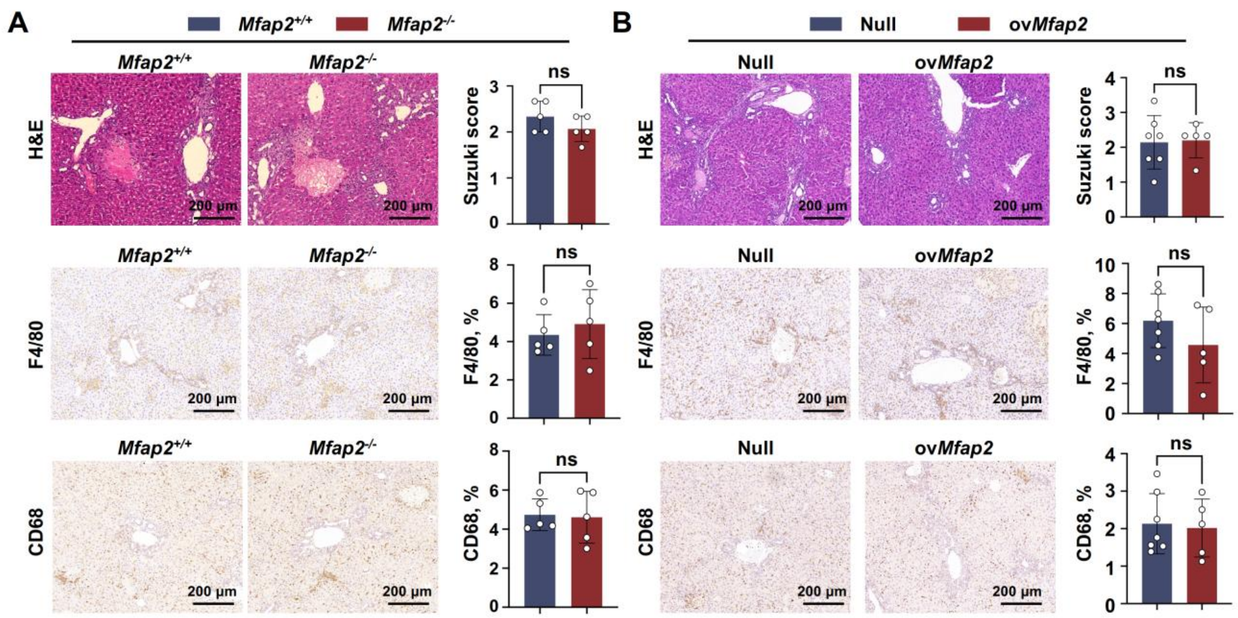

Figure S11. H&E staining and immunostaining analysis of F4/80 or CD68. The Suzuki score based on H&E staining and the percentage of F4/80^{+} and CD68^{+} cells were calculated and compared between (**A**) *Mfap2*$^{+/+}$ and *Mfap2*$^{-/-}$ mice (n = 5/group), or (**B**) Null and ov*Mfap2* mice (n = 5-7/group) post-BDL. Data are expressed as mean ± SEM. ns, not significant.

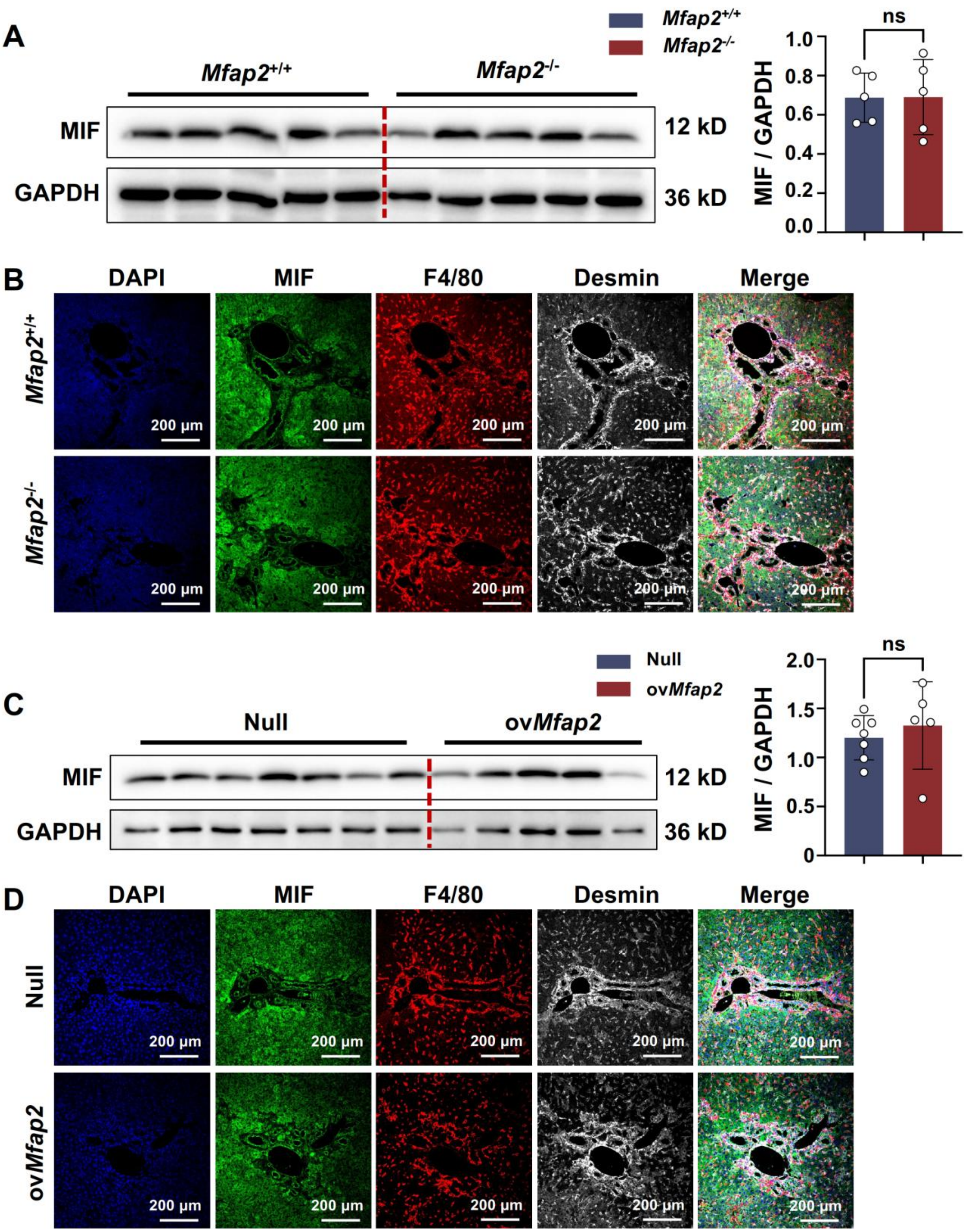

Figure S12. Effects of *Mfap2* ablation or ov*Mfap2* on liver MIF expression in BDL mouse models. (**A, C**) Immunoblotting analysis of MIF in total livers of *Mfap2*$^{-/-}$, *Mfap2*$^{+/+}$, ov*Mfap2,* or Null mice post-BDL (n = 5-7/group). (**B, D**) Multiplex IF staining of MIF (green),

Desmin (red), F4/80 (blue), and DAPI (nucleus, grey) in liver slices of *Mfap2*$^{-/-}$, *Mfap2*$^{+/+}$, ov*Mfap2* or Null mice post-BDL. The merged areas of MIF and Desmin are indicated in yellow. Data are expressed as mean ± SEM. ns, not significant.

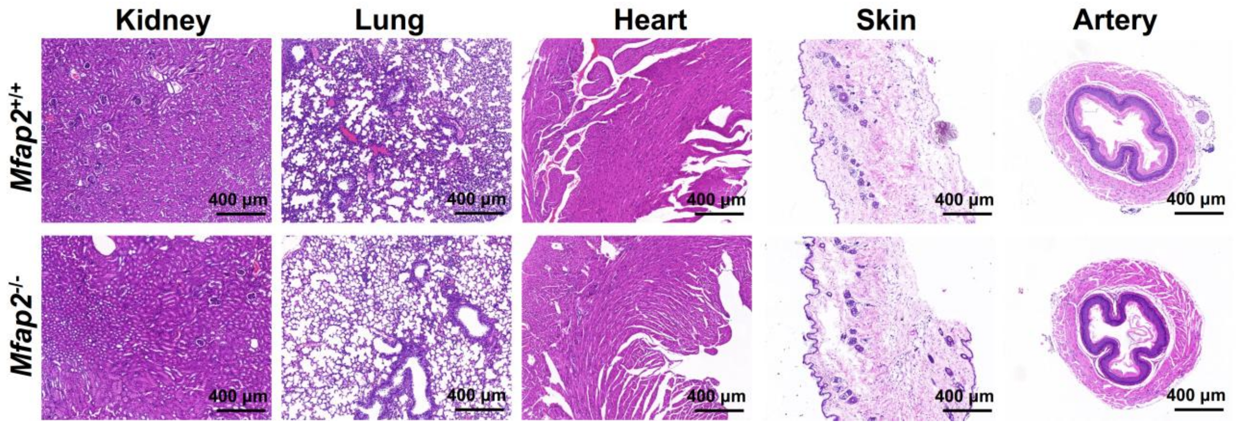

Figure S13. Effects of *Mfap2* deletion on the structure of elastin-rich tissues. H&E staining of kidney, lung, heart, skin, and artery sections from *Mfap2*$^{+/+}$ and *Mfap2*$^{-/-}$ mice.

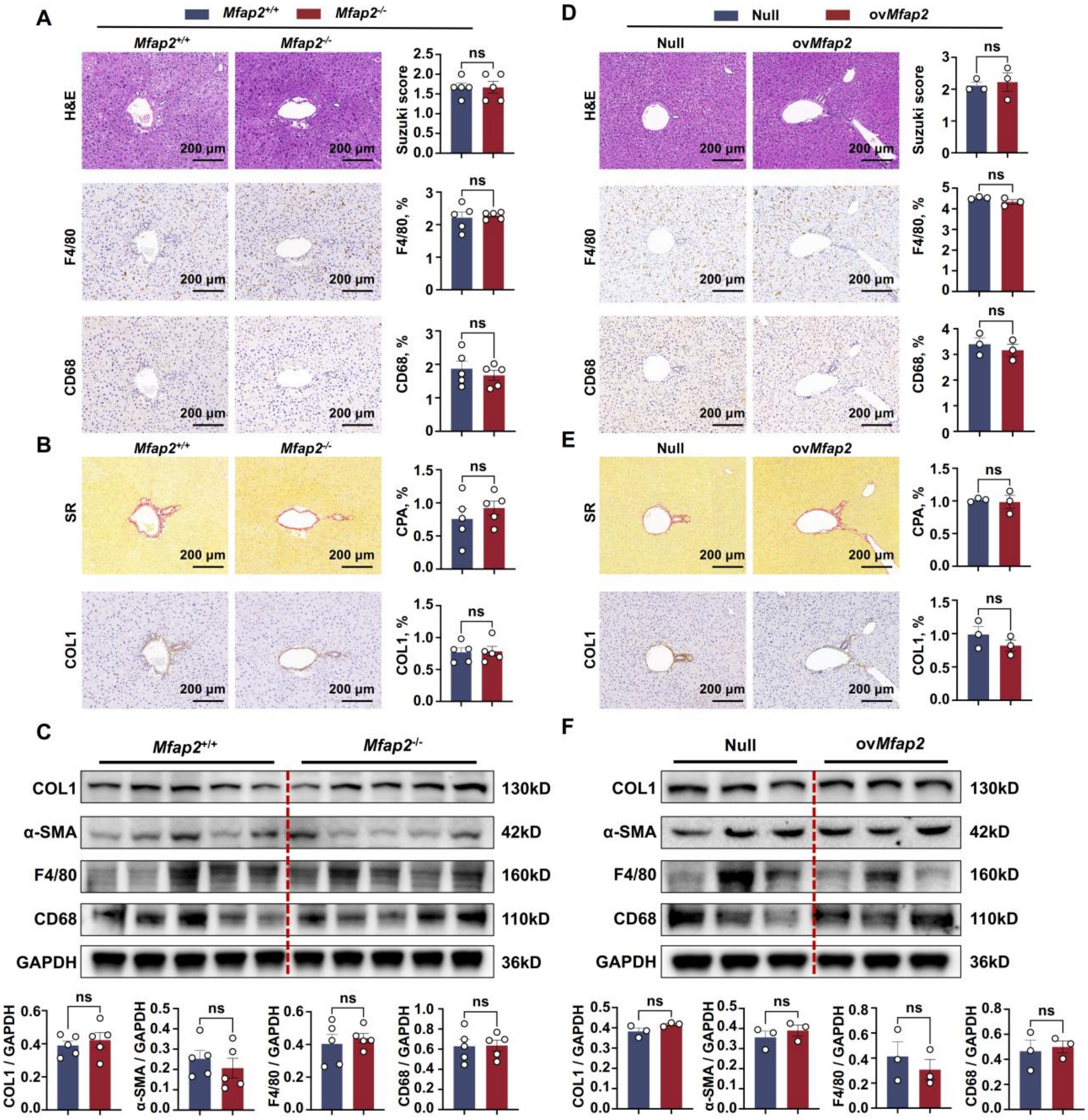

Figure S14. Effects of *Mfap2* intervention on mouse livers in the absence of intoxication. H&E staining and Sirius Red staining of liver sections, along with IHC analysis of F4/80, CD68, and COL1 expression, were performed. Additionally, immunoblotting was conducted to evaluate the protein expression levels of COL1, α-SMA, F4/80, and CD68. Comparisons were made between MO-injected *Mfap2*$^{+/+}$ and *Mfap2*$^{-/-}$ mice (n = 5/group) in panels **A-C**, and between MO-injected Null and ov*Mfap2* mice (n = 3/group) in panels **D-F**.

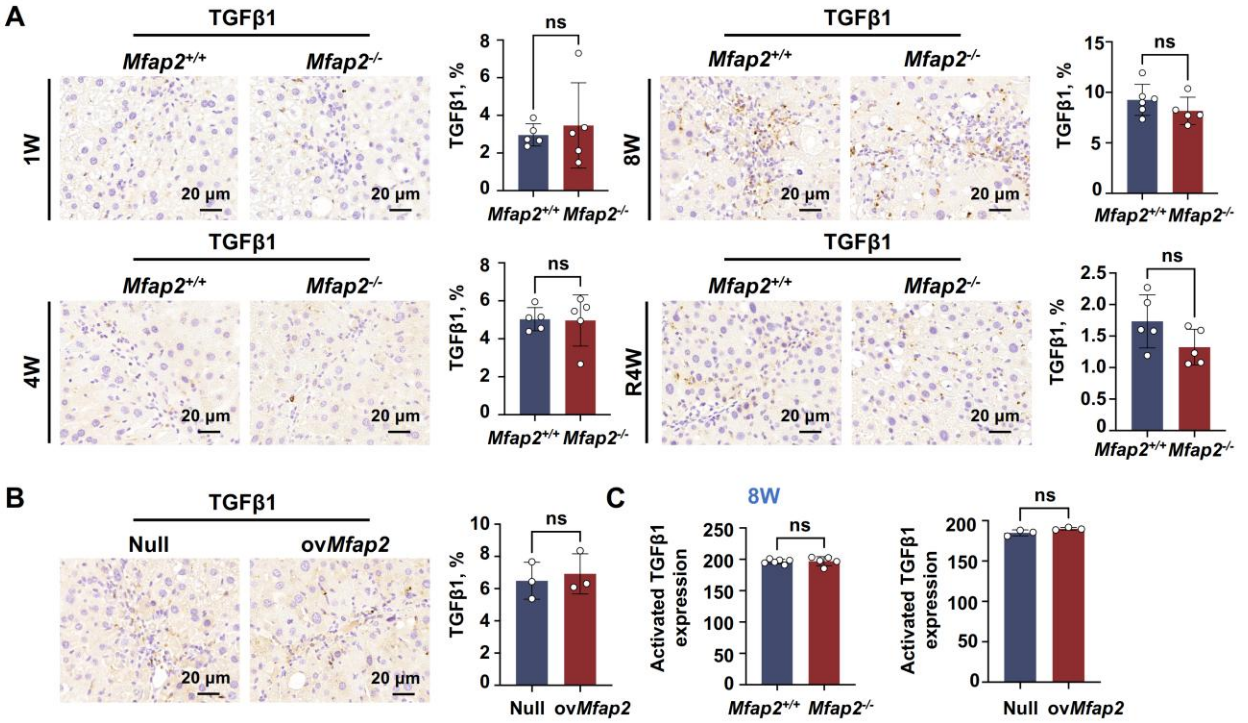

Figure S15. Effects of *Mfap2* deletion or ov*Mfap2* on intrahepatic TGFβ1 expression. (**A**) Immunostaining analysis of TGFβ1 in liver sections between *Mfap2*$^{+/+}$ and *Mfap2*$^{-/-}$ mice after one (1W), four (4W), or eight (8W) weeks of CCl_4 injection, as well as four weeks after CCl_4 cessation (R4W) (n = 5-6/group). (**B**) Immunostaining analysis of TGFβ1 in liver sections between ov*Mfap2* and Null mice after six weeks of CCl_4 injection (n = 3/group). (**C**) Comparison of activated TGFβ1 levels between *Mfap2*$^{+/+}$ and *Mfap2*$^{-/-}$ mice after eight weeks (8W) of CCl_4 injection (n = 5-6/group) or between ov*Mfap2* and Null mice after six weeks of CCl_4 injection (n = 3/group). Data are expressed as mean ± SEM. ns, not significant.

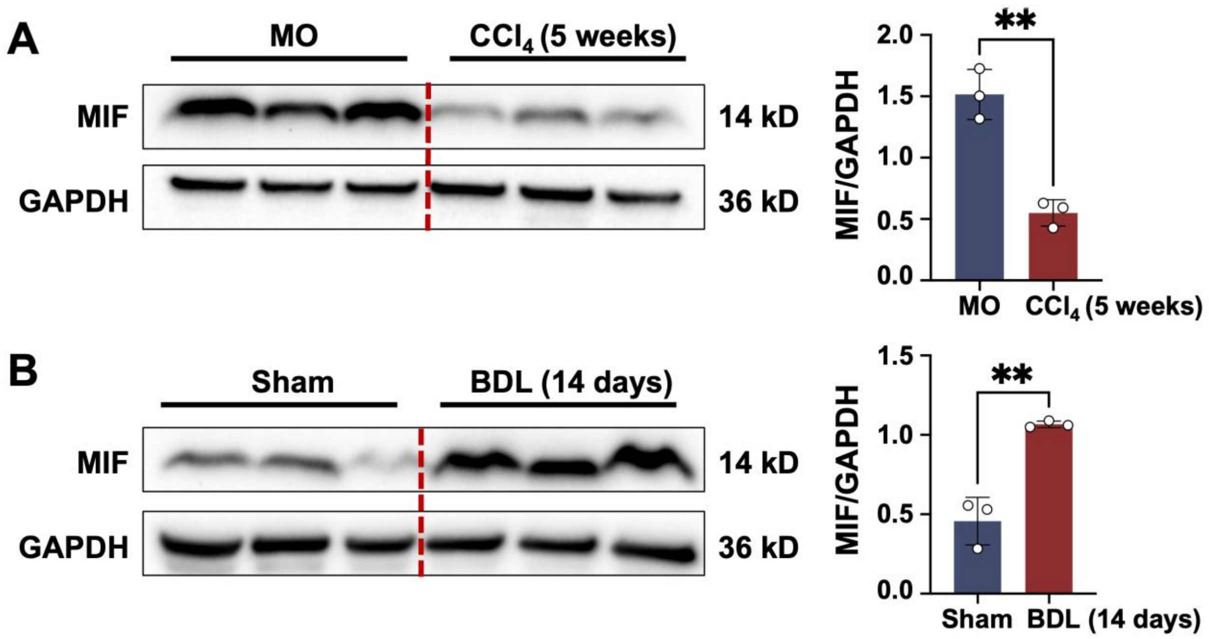

Figure S16. Liver MIF expression in experimental liver fibrogenesis. Immunoblotting analysis of liver MIF in (**A**) CCl_4 (six weeks) or (**B**) BDL (fourteen days) mouse models (n = 3/group). Data are expressed as mean ± SEM. $^{**}p < 0.01$.

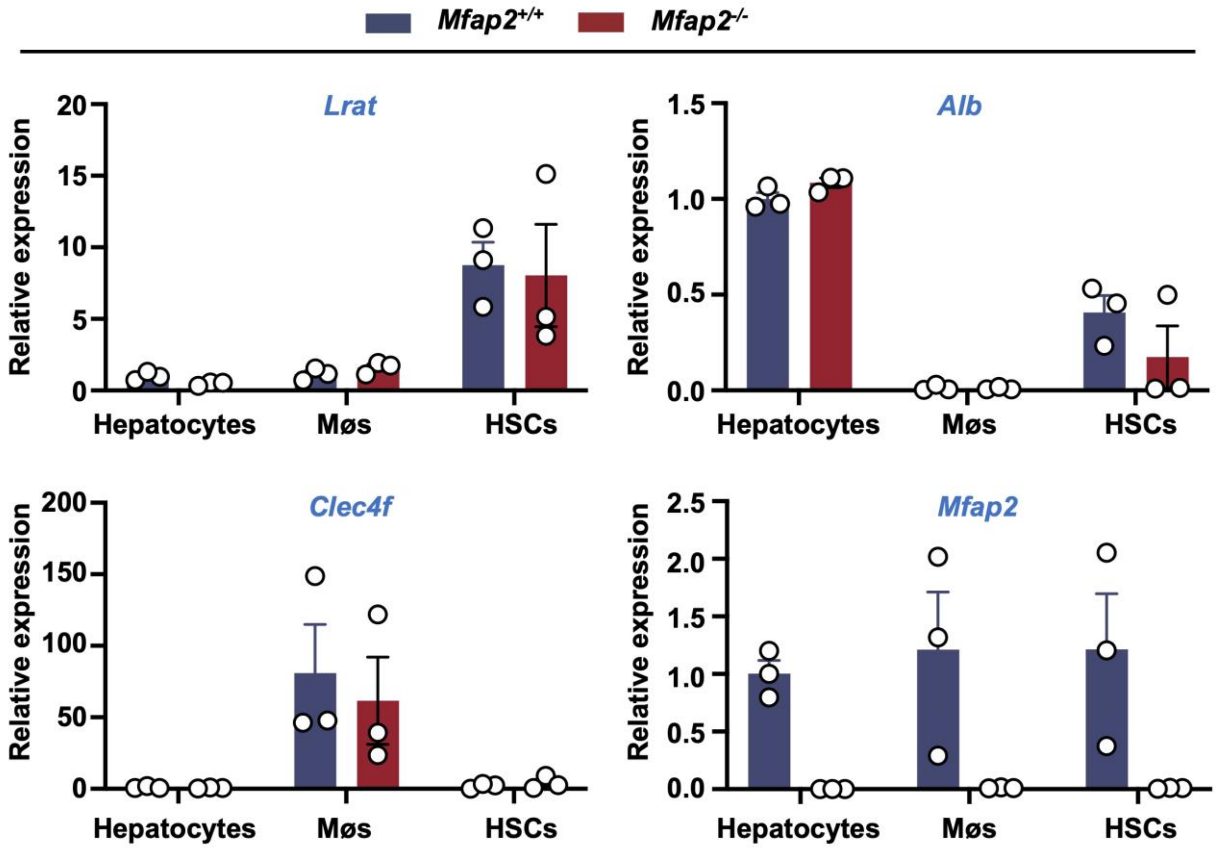

Figure S17. *Mfap2* gene expression in primary liver cells from normal *Mfap2*$^{+/+}$ and *Mfap2*$^{-/-}$ mice. qPCR was conducted to assess the expression levels of *Lrat*, *Alb*, *Clec4f*, and *Mfap2* genes in primary HSCs, hepatocytes, and Møs isolated from both *Mfap2*$^{+/+}$ and *Mfap2*$^{-/-}$ mice without any treatment (n = 3/group, male, 10-month-old). Data are expressed as mean ± SEM.

Supplementary tables

Table S1. Publicly available gene expression profiles of liver fibrosis from the GEO database used in this study.

GEO ID	Etiology	Sample size	Sample resource	Platform	Year
**GSE84044**	HBV	F0 = 43<br>F1 = 20<br>F2 = 33<br>F3 = 18<br>F4 = 10	Frozen liver biopsy	GPL570	2016
**GSE149601**	HCV	Non-LC = 140<br>LC = 55	Liver biopsy	GPL20301	2020
**GSE193066**	MASLD	F0-1 = 51<br>F2 = 71<br>F3-4 = 43	Liver biopsy	GPL18573	2022
**GSE55747**	CCl_4	Con = 4<br>CCl4 = 6	unknown	GPL6885	2017
**GSE74605**	TAA	Con = 3<br>TAA = 3	unknown	GPL6885	2015
**GSE145086**	CCl_4	Control = 3<br>CCl_4 2 weeks = 3<br>CCl_4 4 weeks = 3	NPCs	GPL24247	2020
**GSE233751**	CCl_4	MO = 3<br>Liver fibrosis = 3<br>Resolution = 3	NPCs	GPL24247	2024

GEO: Gene Expression Omnibus; HBV: hepatitis B virus; HCV: hepatitis C virus; LC: liver cirrhosis; MO: mineral oil; non-LC: non-liver cirrhosis; NPCs: non-parenchymal cells; TAA: thioacetamide.

Table S2. List of resources used in this study.

Reagent or resource	Source	Identifier
**Antibodies**		
Anti-CD34 (application: IF/ICC; dilution: 1:100)	Abcam	Cat#ab81289
Anti-CD68 (application: IHC/WB; dilution: 1:100/1:1000)	Thermo Fisher Scientific	Cat#PA5-78996
Anti-COL1 (application: IHC/IF; dilution: 1:100)	CST	Cat#72026
Anti-COL1 (application: WB; dilution: 1:1000)	Thermo Fisher Scientific	Cat#PA5-95137
Anti-COL1 (A2) (application: IF; dilution: 1:100)	Santa Cruz	Cat#sc-393573
Anti-COL3 (application: IF; dilution: 1:100)	Abcam	Cat#ab7778
Anti-Desmin (application: mIF; dilution: 1:500)	Thermo Fisher Scientific	Cat#MA5-32068
Anti-Elastin (application: IF; dilution: 1:100)	Millipore	Cat#MAB2503
Anti-F4/80 (application: IF; dilution: 1:100)	Abcam	Cat#ab6640
Anti-F4/80 (application: mIF/IHC/WB; dilution: 1:100/1:1000)	CST	Cat#70076
Anti-FAK (application: WB; dilution: 1:1000)	CST	Cat#3285
Anti-FBLN-1 (application: IF/WB; dilution: 1:100/1:1000)	ABclonal	Cat#A16677
Anti-FLAG (application: WB; dilution: 1:1000)	Medical & Biological Laboratories	Cat#M185-3L
Anti-GAPDH (application: WB; dilution: 1:10000)	Abcam	Cat#ab8245
Anti-LOXL1 (application: IF; dilution: 1:100)	Santa Cruz	Cat#sc-166632
Anti-LOXL1 (application: WB; dilution: 1:1000)	Thermo Fisher Scientific	Cat#PA5-87701
Anti-LYVE1 (application: IF; dilution: 1:100)	Abcam	Cat#ab281587
Anti-MAGP1 (application: IF; dilution: 1:50)	Santa Cruz	Cat#sc-166075

Anti-MAGP1 (application: IHC; dilution: 1:200)	Novus	Cat#NBP1-87735
Anti-MIF (application: WB/IHC; dilution: 1:1000/1:100)	CST	Cat#87501
Anti-Talin-1 (application: WB/mIF; dilution: 1:1000)	CST	Cat#4021
Anti-Tensin-2 (application: WB; dilution: 1:1000)	CST	Cat#11990
Anti- TGFβ1 (application: IHC; dilution: 1:1000)	Thermo Fisher Scientific	Cat#MA-21595
Anti-Vinculin (application: WB; dilution: 1:1000)	CST	Cat#4650
Anti-α-Actinin (application: WB; dilution: 1:1000)	CST	Cat#6487
Anti-α-SMA (application: IF/mIF; dilution: 1:100)	Abcam	Cat#ab7817
Anti-α-SMA (application: WB; dilution: 1:1000)	Abcam	Cat#ab5694
Alexa Fluor 488 donkey anti-rabbit IgG(H+L) (application: secondary antibody; dilution: 1:500)	Invitrogen	Cat#A21206
Alexa Fluor 488 donkey anti-mouse IgG(H+L) (application: secondary antibody; dilution: 1:500)	Invitrogen	Cat#A21202
Alexa Fluor 594 donkey anti-rabbit IgG(H+L) (application: secondary antibody; dilution: 1:500)	Invitrogen	Cat#A21207
Alexa Fluor 594 donkey anti-mouse IgG(H+L) (application: secondary antibody; dilution: 1:500)	Invitrogen	Cat#A21203
Alexa Fluor 594 donkey anti-rat IgG(H+L) (application: secondary antibody; dilution: 1:500)	Invitrogen	Cat#A21209
Anti-mouse IgG HRP-linked Ab (application: secondary antibody; dilution: 1:5000)	ZSGB-Bio	Cat#ZB-2305

Anti-rabbit IgG HRP-linked Ab (application: secondary antibody; dilution: 1:5000)	ZSGB-Bio	Cat#ZB-2301
**Chemicals, peptides, commercial kits, and recombinant proteins**		
4',6-diamidino-2-phenylindole	Abcam	Cat#ab104139
Acetone	Sinopharm Chemical Reagent Co., Ltd	Cat#10000418
Acetonitrile	Sigma	Cat#34851
Agilent High Sensitivity DNA Kit	Agilent	Cat#5067-4626
Alanine Aminotransferase Assay Kit	Nanjing Jiancheng	Cat# C009-2-1
Ammonium bicarbonate	Sigma	Cat#09830
Aspartate Aminotransferase Assay Kit	Nanjing Jiancheng	Cat# C010-2-1
Carbon tetrachloride	Innochem	Cat#A68354
Chromium Single Cell 3' Library & Single Cell 3' v3 Gel Beads	10× Genomics	Cat#PN-1000075
Citrate buffer	ZSGB-BIO	Cat#ZLI-9064
DAB HRP Substrate Kit	ZSGB-BIO	Cat#ZLI-9018
Dithiothreitol	Thermo Fisher Scientific	Cat#20290
DynaBeads® MyOneTM Silane Beads	Life Technologies	Cat#37002D
Fast Mouse Genotyping Kit	Beyotime	Cat#D7283S
Fetal bovine serum	Sigma	Cat#F8687-500ml
Formic acid	Sigma	Cat#27001
H&E Staining Kit	Solarbio	Cat# G1120
Human liver tissue array	US Biomax, Inc	Cat# LV805b

Hydrogen peroxide (3%)	Sinopharm Chemical Reagent Co., Ltd	Cat#10011208
Illumina VAHTS® Universal V6 RNA-seq Library Prep Kit	Vazyme	Cat#V6-003
Iodoacetamide	Thermo Fisher Scientific	Cat#ICN1003510 5
LEGEND MAX™ Free Active TGFβ1 ELISA Kit	Biolegend	Cat# No.437707
Lys-C	Wako	Cat#125-05061
Magnetic oligo (dT) beads	Invitrogen	Cat#61002
Minimum Essential Medium	Procell	Cat#PM150411
Mineral oil	Thermo Fisher Scientific	Cat#J62592.AP
Modified Sirius Red Staining Kit	Solarbio	Cat#G1472
Mouse Liver Dissociation Kit	Miltenyi Biotec	Cat#130-105-807
NaCl	Sigma	Cat#S9888
NEBNext Ultra RNA Library Prep Kit	New England Biolabs	Cat#E7770
Nycodenz	AXELL	Cat#AN1002424
OCT compound	Sakura	Cat#4583
PANO 4-plex IHC kit	Panovue	Cat#TSA-RM-275
Penicillin and streptomycin	Gibco	Cat#15140122
Percoll	Cytiva	Cat#17089109
Pierce BCA Protein Assay Kit	Thermo Fisher Scientific	Cat#23227
Pierce™ Protein Concentrator PES 10K MWCO, 2-6 mL, 24PK	Thermo Fisher Scientific	Cat#88517
PNGaseF	BioLabs	Cat#P0704L
PrimeScript™ RT reagent kit	Takara	Cat#RR037Q
Protease inhibitor	Yeasen	Cat#20124ES03

Qubit dsDNA Assay Kit	Life Technologies	Cat#Q328520
rhTGFβ1	MCE	Cat#HY-P7118
RNA simple Total RNA kit	Fastagen	Cat#220010
RPMI-1640 Complete Medium	Procell	Cat#PM150110B
SDS	Sigma	Cat#L3771
SPRIselect Reagent Kit	Life Technologies	Cat#B23318
Trifluoroacetic acid	Thermo Fisher Scientific	Cat#A116-10X1 AMP
Tris base	Solarbio	Cat#T8060
Triton X-100	Solarbio	Cat#T8200
Trypsin	Promega	Cat#V511A
Urea	Sigma	Cat#U6504
X-tremeGENE HP DNA Transfection Reagent	Roche	Cat#6366236001
**Cell lines**		
Human: LX-2 cell line	ATCC	N/A
Human: THP-1 cell line	Procell	CL-0233
**Organisms/strains**		
Mouse: C57BL/6J	HFK Bioscience Co. Ltd.	N/A
Mouse: *Mfap2*$^{-/-}$ and *Mfap2*$^{+/+}$	Cyagen Bioscience Inc.	S-KO-03113
**Primers for qPCR and genotyping**		
Human_*MFAP2*_forward:<br>5' – ACCCGCCTCTACTCCATACA – 3'<br>Human_*MFAP2*_reverse:<br>5' – TAATGACGTACACACGGCGG – 3'	This paper	Designed by Primer-BLAST
Human_*MIF*_forward:<br>5' – CTGCACAGCATCGGCAAGAT – 3'<br>Human_ *MIF*_reverse:<br>5' – AGTTGATGTAGACCCTGTCCG – 3'	This paper	Designed by Primer-BLAST

Human_*ADGRE1*_forward:<br>5' – CCAGTGTTAATGCCGAAGTCT – 3'<br>Human_ *ADGRE1*_reverse:<br>5' – GTGAACAGGTAAGCCATGACA - 3'	This paper	Designed by Primer-BLAST
Human_*CD163*_forward:<br>5' – TTTGTCAACTTGAGTCCCTTCAC – 3'<br>Human_*CD163*_reverse:<br>5' – TCCCGCTACACTTGTTTTCAC – 3'	This paper	Designed by Primer-BLAST
Human_*CLEC4F*_forward:<br>5' – CCCCAAGATACCGAGGCTC – 3'<br>Human_*CLEC4F*_reverse:<br>5' – GCCCAGTAATGTTGTCTCCCA – 3'	This paper	Designed by Primer-BLAST
Human_*CX3CR1*_forward:<br>5' – ACTTTGAGTACGATGATTTGGCT – 3'<br>Human_*CX3CR1*_reverse:<br>5' – GGTAAATGTCGGTGACACTCTT – 3'	This paper	Designed by Primer-BLAST
Human_*GAPDH*_forward:<br>5' – GGAGCGAGATCCCTCCAAAAT – 3'<br>Human_ *GAPDH*_reverse:<br>5' – GGCTGTTGTCATACTTCTCATGG – 3'	This paper	Designed by Primer-BLAST
Mouse_*Alb*_forward:<br>5' – TGCTTTTTCCAGGGGTGTGTT – 3'<br>Mouse_*Alb*_reverse:<br>5' – TTACTTCCTGCACTAATTTGGCA – 3'	This paper	Designed by Primer-BLAST
Mouse_*Lrat*_forward:<br>5' – CCGTCCCTATGAAATCAGCTC – 3'<br>Mouse_*Lrat*_reverse:<br>5' – ATGGGCGACACGGTTTTCC – 3'	This paper	Designed by Primer-BLAST
Mouse_*Clec4f*_forward:<br>5' – GAGGCCGAGCTGAACAGAG – 3'<br>Mouse_*Clec4f*_reverse:	This paper	Designed by Primer-BLAST

5’ – TGTGAAGCCACCACAAAAAGAG – 3’		
Mouse_*Tnf*_forward:<br>5’ – GACGTGGAACTGGCAGAAGAG – 3’<br>Mouse_*Tnf*_reverse:<br>5’ – TTGGTGGTTTGTGAGTGTGAG – 3’	This paper	Designed by Primer-BLAST
Mouse_*Il1b*_forward:<br>5’ – GCAACTGTTCCTGAACTCAACT – 3’<br>Mouse_*Il1b*_reverse:<br>5’ – ATCTTTTGGGGTCCGTCAACT – 3’	This paper	Designed by Primer-BLAST
Mouse_*Il6*_forward:<br>5’ – CCAAGAGGTGAGTGCTTCCC – 3’<br>Mouse_*Il6*_reverse:<br>5’ – CTGTTGTTCAGACTCTCTCCCT – 3’	This paper	Designed by Primer-BLAST
Mouse_*Ccl2*_forward:<br>5’ – TTAAAAACCTGGATCGGAACCAA – 3’<br>Mouse_*Ccl2*_reverse:<br>5’ – GCATTAGCTTCAGATTTACGGGT – 3’	This paper	Designed by Primer-BLAST
Mouse_*Mfap2*$^{+/+}$_forward:<br>5’ – TCACCAAGACCACACTCTTGTTA – 3’<br>Mouse_*Mfap2*$^{+/+}$_reverse:<br>5’ – CGACCTCTCTAAGAGCCACTTG – 3’	This paper	Cyagen Bioscience Inc.
Mouse_*Mfap2*$^{-/-}$_forward:<br>5’ – TCACCAAGACCACACTCTTGTTA – 3’<br>Mouse_*Mfap2*$^{-/-}$_reverse:<br>5’ – ATTACATAGACCTGTGAGGAGGGAC – 3’	This paper	Cyagen Bioscience Inc.
Mouse_*Mfap2*_forward:<br>5’ – CGCCGAGTGTATGTGGTCAA – 3’<br>Mouse_*Mfap2*_reverse:<br>5’ – ACGCCACACTTGGAgAACTT – 3’	This paper	Designed by Primer-BLAST
Mouse_*Gapdh*_forward:<br>5’ – TGGCCTTCCGTGTTCCTAC – 3’	This paper	Designed by Primer-BLAST

Mouse_*Gapdh*_reverse:<br>5’ – GAGTTGCTGTTGAAGTCGCA – 3’		
**AAV vectors, plasmids, and siRNAs**		
pRP[Exp]-EGFP/Puro-EF1A>hMFAP2/FLAG plasmid	VectorBuilder	Cat#VB230216-1551xdw
pRP[Exp]-EGFP/Puro-EF1A>ORF_Stuffer (null)	VectorBuilder	Cat#VB180929-1003hrg
Human *MFAP2* siRNA<br>Target sequence: ACUGUACGAACACAGAUCUCCTTP	OBiO	Cat#MFAP2-135-A
Negative control siRNA<br>Target sequence: ACGUGACACGUUCGGAGAATT	OBiO	NA
AAV6-CMV-Mfap2-HA-EF1a-mNeonGreen-3×FLAG-WPRE vector	OBiO	Cat#H23692
AAV6-CMV-MCS-EF1a-mNeonGreen-3×FLAG-WPRE vector	OBiO	Cat#H10018
**Software**		
7500 Software v2.3	Applied Biosystems	https://www.thermofisher.cn/cn/zh/home/brands/applied-biosystems.html
CellChat	Suoqin Jin et al.	https://github.com/sqjin/CellChat
Cufflinks	Cole Trapnell’s lab	http://cole-trapnell-lab.github.io/cufflinks/

DAVID web tool	Brad T Sherman et al.	https://david.ncifcrf.gov/
DoubletFinder v2.0.2	Christopher S McGinnis et al.	https://github.com/chris-mcginnis-ucsf/DoubletFinder
FV10-ASW 4.2 Viewer	Olympus Life Science	https://www.olympus-lifescience.com
Gene Expression Omnibus	National Institutes of Health	https://www.ncbi.nlm.nih.gov/geo/
ggplot2	-	https://cran.r-project.org/web/packages/ggplot2/index.html
GraphPad Prism version 9	Dotmatics	https://www.graphpad.com
HISAT software	Daehwan Kim et al.	https://github.com/infphilo/hisat
ImageJ	the National Institutes of Health	https://imagej.net/ij/
Image Lab Software	Bio-Rad	https://www.bio-rad.com/
Image-Pro Plus software version 6.0	Media Cybernetics	https://image-pro-plus.software.informer.com/6.0/
mMCPcounter	Florent Petitprez et al.	https://github.com/cit-bioinfo/mMCP-counter

Primer-BLAST	National Institutes of Health	https://www.ncbi.nlm.nih.gov/tools/primer-blast
Proteome Discoverer suite version 2.4	Thermo Fisher Scientific	https://www.thermofisher.cn/cn/zh/home.html
ProteomeXchange consortium	Juan A Vizcaíno et al.	https://www.proteomexchange.org/
R 4.2.1	-	https://www.r-project.org/
Rstudio	-	https://posit.co/products/open-source/rstudio/#
**Other**		

Table S3. Comparison of liver index and blood biochemistry between *Mfap2*$^{+/+}$ and *Mfap2*$^{-/-}$ mice or Null and ov*Mfap2* mice undergoing CCl_4 injection or BDL operation.

	Group	LW/BW ratio	ALT, U/L	AST, U/L
**CCl_4 (1W)**	***Mfap2*$^{+/+}$**	0.06±0.00	95.16±13.66	33.86±3.59
	***Mfap2*$^{-/-}$**	0.06±0.00	122.40±7.71	55.43±21.47
	***p* value**	0.466	0.121	0.351
**CCl_4 (4W)**	***Mfap2*$^{+/+}$**	0.04±0.00	121.90±10.36	90.16±2.36
	***Mfap2*$^{-/-}$**	0.04±0.00	108.40±8.53	82.40±2.44
	***p* value**	0.551	0.343	0.052
**CCl_4 (8W)**	***Mfap2*$^{+/+}$**	0.04±0.00	139.50±1.44	76.01±2.89
	***Mfap2*$^{-/-}$**	0.05±0.00	130.50±5.08	65.79±4.22
	***p* value**	0.277	0.098	0.070

**CCl_4 (R4W)**	***Mfap2*$^{+/+}$**	0.06±0.01	41.92±18.41	65.02±14.18
	***Mfap2*$^{-/-}$**	0.06±0.00	29.19±6.32	45.78±9.62
	***p* value**	0.796	0.531	0.294
**CCl_4 (6W)**	**Null**	0.05±0.00	138.50±5.11	122.10±36.54
	**ov*Mfap2***	0.04±0.00	131.30±4.27	77.04±1.40
	***p* value**	0.224	0.339	0.285
**CCl_4 (R3W)**	**Null**	0.06±0.00	13.18±3.10	23.36±1.05
	**ov*Mfap2***	0.06±0.00	8.70±1.45	14.20±1.19
	***p* value**	0.837	0.215	0.000
**BDL (14D)**	***Mfap2*$^{+/+}$**	0.59±0.02	36.27±2.39	31.21±7.56
	***Mfap2*$^{-/-}$**	0.58±0.02	43.43±5.16	31.77±2.47
	***p* value**	0.705	0.243	0.946
**BDL (14D)**	**Null**	0.65±0.02	32.66±6.25	41.90±7.96
	**ov*Mfap2***	0.62±0.03	30.07±7.31	41.47±6.71
	***p* value**	0.413	0.793	0.970

LW/BW ratio: liver to body weight ratio.
